# Combined frequency-tagging EEG and eye-tracking measures provide no support for the “excess mouth/diminished eye attention” hypothesis in autism

**DOI:** 10.1186/s13229-020-00396-5

**Published:** 2020-11-23

**Authors:** Sofie Vettori, Stephanie Van der Donck, Jannes Nys, Pieter Moors, Tim Van Wesemael, Jean Steyaert, Bruno Rossion, Milena Dzhelyova, Bart Boets

**Affiliations:** 1 Center for Developmental Psychiatry, Department of Neurosciences, University of Leuven (KU Leuven), Leuven, Belgium; 2 Leuven Autism Research (LAuRes), University of Leuven (KU Leuven), Leuven, Belgium; 3 Institute of Research in Psychological Science, Institute of Neuroscience, University of Louvain, Louvain-La-Neuve, Belgium; 4 Department of Physics and Astronomy, Ghent University, Ghent, Belgium; 5 IDLab - Department of Computer Science, University of Antwerp - IMEC, Antwerp, Belgium; 6 Laboratory of Experimental Psychology, University of Leuven (KU Leuven), Leuven, Belgium; 7 Department of Electrical Engineering (ESAT), Stadius Center for Dynamical Systems, Signal Processing and Data Analytics, Leuven, Belgium; 8 CNRS, CRAN - UMR 7039, Université de Lorraine, 54000 Nancy, France; 9 CHRU-Nancy, Service de Neurologie, Université de Lorraine, 54000 Nancy, France

## Abstract

**Background:**

Scanning faces is important for social interactions. Difficulty with the social use of eye contact constitutes one of the clinical symptoms of autism spectrum disorder (ASD). It has been suggested that individuals with ASD look less at the eyes and more at the mouth than typically developing (TD) individuals, possibly due to gaze aversion or gaze indifference. However, eye-tracking evidence for this hypothesis is mixed. While gaze patterns convey information about overt orienting processes, it is unclear how this is manifested at the neural level and how relative covert attention to the eyes and mouth of faces might be affected in ASD.

**Methods:**

We used frequency-tagging EEG in combination with eye tracking, while participants watched fast flickering faces for 1-min stimulation sequences. The upper and lower halves of the faces were presented at 6 Hz and 7.5 Hz or vice versa in different stimulation sequences, allowing to objectively disentangle the neural saliency of the eyes versus mouth region of a perceived face. We tested 21 boys with ASD (8–12 years old) and 21 TD control boys, matched for age and IQ.

**Results:**

Both groups looked longer at the eyes than the mouth, without any group difference in relative fixation duration to these features. TD boys looked significantly more to the nose, while the ASD boys looked more outside the face. EEG neural saliency data partly followed this pattern: neural responses to the upper or lower face half were not different between groups, but in the TD group, neural responses to the lower face halves were larger than responses to the upper part. Face exploration dynamics showed that TD individuals mostly maintained fixations within the same facial region, whereas individuals with ASD switched more often between the face parts.

**Limitations:**

Replication in large and independent samples may be needed to validate exploratory results.

**Conclusions:**

Combined eye-tracking and frequency-tagged neural responses show no support for the excess mouth/diminished eye gaze hypothesis in ASD. The more exploratory face scanning style observed in ASD might be related to their increased feature-based face processing style.

## Introduction

### The dominance of gaze fixation on the eyes of faces

Scanning faces of conspecifics with eye movements is important for social interactions in our species. An important carrier of socially relevant information is the mouth, which is the main source of visual information relevant to speech [35]. When auditory cues are less informative (e.g., when environmental noise increases), the proportion of gaze fixations on the mouth increases [10, 95]. Developmental work of Lewkowicz and Hansen-Tift [51] has shown that when learning language, around 4–8 months of age, infants temporarily look more at the mouth of videotaped faces, presumably to pick up (redundant) audiovisual information. Yet, the overwhelming majority of studies have shown that when scanning faces, people often look first and foremost towards the eyes [6, 34, 66, 100, 107]. Preferential fixation to the eyes of conspecifics’ faces is important since maintaining good eye contact carries significant social value [35]. Moreover, people move their gaze to fixate locations on the face that maximize their recognition of, for example, identity, gender, or emotional state of people. In neurotypical observers, the optimal fixation location for a variety of face recognition/categorization functions has been identified as a particular (featureless) central point just below the eyes, at the nasion [40, 67, 105], which is hypothesized as the “center of mass of the face” allowing to grasp all of its diagnostic features at once (“holistic/configural face perception” [77, 105]. At a group level, deviations from this optimal fixation point have been associated with substantial detriment of face processing performance [67]. Nevertheless, there is both cultural [7] and interindividual variability in the exact position of this optimal fixation point [53, 68, 87], and each individual’s looking preference corresponds to an idiosyncratic performance-maximizing point of fixation.

### “Excess mouth/diminished eye gaze” hypothesis in autism

Individuals with ASD are characterized by impairments in social communication and interaction, combined with a pattern of restricted and repetitive behavior and interests [2]. Abnormalities in the social use of eye contact constitute one of the symptoms of the socio-communicative symptom domain of the DSM-5 [2]. A seminal eye-tracking study reported that adolescents and adults with ASD look relatively less at the eyes and more to the mouth than matched typically developing (TD) individuals [48]. Moreover, those individuals from the ASD group that did attend more to the mouth region had better social abilities than those that did not attend to the face at all, suggesting that attending to the mouth could be seen as a compensatory mechanism. This pioneering study attracted a lot of attention and resulted in the so-called excess mouth/diminished eye gaze hypothesis in ASD [48], which was indirectly supported by face-processing literature showing that individuals with ASD have particular difficulties discriminating the eyes and therefore rely preferentially on mouth information to individuate faces [44, 81, 86, 104]. Further research showed that the amount of time spent looking at the eye region correlated with brain activation in the face-selective region of the fusiform gyrus in individuals with ASD [22], thereby suggesting that diminished fixation on the eye region may account for the reported fusiform gyrus hypoactivation in ASD [21, 63, 70].

However, while some studies confirmed that adults with ASD look less at the eye region of faces (e.g., [17, p. 200, 58, 85]), others did not (e.g., [28, 82]). Altogether, while the “excess mouth/diminished eye gaze” hypothesis has been highly influential in the clinical and scientific field of ASD, a series of formal meta-analyses of empirical studies across all ages have found little evidence for it [27, 30, 31, 64], and a number of factors have been proposed to account for discrepancies in observations across studies: degree of symptom severity, small and heterogeneous samples, differences in outcome measures, and differences in the content of the applied stimuli (e.g., dynamic face stimuli involving social interactions versus static face stimuli). To minimize such confounds, Kwon et al. recently conducted an eye-tracking study in a large sample of toddlers with and without ASD (N = 385) [50]. Across two experiments, typical levels of eye and mouth looking were found in toddlers with ASD as compared to the control group, in line with the results of the meta-analyses. Overall, and particularly when a geometric distractor was present, toddlers with ASD showed decreased fixation time to the overall face.

Another important issue is that visual scanning patterns are likely to change with age. As a result, group differences in face scanning patterns may be age dependent. Indeed, among the studies investigating face scanning in children with ASD younger than 12 years, only one has found evidence for the “excess mouth/diminished eyes” hypothesis [42]. Other studies did not find any difference between face parts scanned in ASD and neurotypical observers [23, 26, 50, 94], and some studies even reported longer looking times to the mouth in the control group than in the ASD group [14, 58].

Rather than investigating looking times to particular parts of the face or the visual scene, more recent studies started analyzing and modeling the temporal scan paths in order to obtain a more comprehensive measure of the face exploration dynamics (e.g., [19, 20]), for instance by using Markov models. Along these lines, Vabalas and Freeth [91] showed that adults with high autistic traits exhibit reduced visual exploration during face-to-face interactions. Likewise, Heaton and Freeth [32] showed that adolescents with ASD demonstrated less exploration of photographic scenes, both when the scene contained a central face or not. Moreover, the participants with ASD showed a greater tendency to explore areas close to the current fixation. These studies underline the importance of analyzing data beyond mere fixation duration in order to pinpoint also the more subtle face exploration dynamics.

### Gaze avoidance versus gaze indifference

Two hypotheses are often put forward to explain why individuals with ASD may attend less to the eyes: gaze avoidance on the one hand and gaze indifference on the other. The gaze avoidance hypothesis proposes that the eye region is perceived as aversive and socially threatening by individuals with ASD, as indicated by heightened skin conductance and amygdala reactivity in response to facial stimuli [88]. The gaze indifference hypothesis, on the other hand, should be situated against the background of the social motivation theory of autism and implies that the eye region is not as socially relevant for individuals with ASD as it is for neurotypical controls [15]. However, both explanations are not necessarily contradictory and could be embedded in a common developmental trajectory. An early lack of interest in eye contact may cause children with autism to miss out on social cues, leading to low social motivation and interest later on [55]. Consequently, having to engage in eye contact while not being socially motivated may feel unpleasant and aversive for them, which may result in the active avoidance of eye contact in some older individuals with ASD [47]. Alternatively, gaze avoidance may precede gaze indifference: Social stimuli might be less intrinsically rewarding because they are experienced as over-arousing.

### Frequency-tagging EEG as a complementary measure to assess the “excess mouth/diminished eye gaze” hypothesis

An intriguing possibility is that individuals with ASD do not differ reliably in their overt attention to the eyes and mouth of a face as compared to neurotypical individuals, but these two groups nevertheless differ in the amount of covert attention devoted to these facial parts. Unfortunately, while eye tracking informs about overt orienting behavior, it precludes measuring the processing of stimuli outside the focus of overt attention. Yet, the neural intensity of stimulus processing, both inside and outside the focus of attention, can be captured by means of electroencephalography (EEG), if these stimuli are “labeled” (i.e., dissociated in time, space or frequency) appropriately [38, 39]. In the present study, we combine simultaneous eye tracking and EEG frequency-tagging to capture both overt and covert processing of the eye and mouth region of neutral faces in children with ASD and typically developing controls. The frequency-tagging EEG technique is based on the fairly old observation that a visual stimulus presented at a fixed rate, e.g., a light flickering on/off 17 times per second (17 Hz), generates an electrical brain wave exactly at the stimulation frequency (i.e., 17 Hz in this example), which can be recorded over the visual cortex [1]. By transforming the data in the frequency domain through Fourier analysis [72], a highly *sensitive* (i.e., high signal-to-noise ratio, SNR) [73] and *objective* (i.e., at a predetermined frequency) quantifiable marker of automatic visual processes without explicit task demands is provided. Moreover, by assigning different tags (frequencies) to different stimuli in a multi-input stimulation, the respective responses corresponding to each of the stimulation frequencies can be disentangled (“frequency-tagging”, [74]). Hence, evoked responses from populations of cells that are selective to each of the unique input stimuli can be extracted and quantified, even if input stimuli are spatially overlapping, embedded within the same stimulus event or outside of the focus of attention [60].

Importantly, changes in amplitude of the neural responses represent dynamic neural changes related to the processing of the driving stimuli and are modulated by attention [3, 54, 56, 90, 99], memory or emotion [89]. In a recent study [24], the eye and mouth region of faces were frequency-tagged, while EEG and MEG signals were recorded. By combining the frequency-tagged MEG responses with functional ROIs defined from fMRI, the researchers found that changes in both face parts (eyes and mouth) resulted in enhanced responses in a face-selective region of the inferior occipital gyrus, while the superior temporal sulcus (STS) was mostly responsive to changes in the eye region. Moreover, top-down attention to the eyes versus the mouth (while maintaining central fixation) resulted in enhanced neural processing in the respective brain area.

We recently assessed social preference in boys with and without ASD by simultaneously measuring EEG responses and looking times for frequency-tagged streams of social (faces) versus non-social (houses) stimuli. In particular, we demonstrated a reduced social bias in boys with ASD, and we found that group differences in the relative preference for social information were far more pronounced in the frequency-tagged neural responses than in the looking times assessed by eye tracking [98].

Against this background, the present study was designed to compare overt and covert attention in children with autism (ASD) and a matched typically developing control group (TD) by presenting facial stimuli which where horizontally subdivided along the nasion, displaying the eyes versus mouth stimulus regions at different presentation rates. Hence, we were able to objectively disentangle the frequency-tagged neural responses to the eye region versus the mouth region.

The aims of the current study are fourfold. First, the neural saliency of processing the eyes versus mouth region can be objectively quantified via EEG frequency-tagging. Second, eye-tracking measures allow investigating potential group differences in looking times to the eyes, mouth and nose region. Third, subtle face exploration dynamics are captured by modeling the temporal fixation scanpaths. Fourth, the relation between overt attention to particular facial parts (as measured with eye-tracking) and covert brain responses to these facial features (as measured with EEG) will be investigated. This integration of overt and covert measures may allow determining whether the eyes are perceived as too aversive, or whether they are rather experienced as less relevant by individuals with ASD. In particular, aversive stimuli are expected to elicit large neural responses even if they may be unattended or actively avoided, whereas less relevant stimuli are expected to be less fixated and elicit lower neural frequency-tagged responses.

## Material and methods

We recruited 47 boys, aged 8 to 12 years old. This age range was chosen to capture a large developmental window at school age. Data from one participant were lost due to technical issues during EEG recording. To match the groups on verbal and performance IQ (VIQ, PIQ), four participants (two from the TD group and two from the ASD group) were excluded from the reported analyses, resulting in a sample of 21 typically developing (TD) boys (mean age = 10.2 years ± SD = 1.3) and 21 boys with ASD (mean age = 10.6 ± 1.3, Table 1). However, inclusion of these four participants did not change any of the reported results of the analyses. The sample in this study is identical to the one in previous studies [97, 98] where frequency-tagged social and non-social stimuli were presented. All participants had normal or corrected-to-normal vision and had a verbal and performance IQ above 80. Thirty-nine participants were right-handed. Participants with ASD were recruited through the Autism Expertise Center of the University Hospitals Leuven, Belgium. TD participants were recruited through elementary schools and sports clubs.

**Table 1:** Participant characteristics

	ASD (mean ± SD)	TD (mean ± SD)
Verbal IQ	107 ± 11	112 ± 13
Performance IQ	105 ± 15	111 ± 14
Age	10.6 ± 1.3	10.21 ± 1.3
Social Responsiveness Scale (*T*-score)	85 ± 12	42 ± 6

Participant exclusion criteria were the presence or suspicion of a psychiatric, neurological, learning or developmental disorder (other than ASD or comorbid ADHD in ASD participants, which was the case in 5 participants) in the participant or in a first- or second-degree relative. Exclusion of these participants with comorbid ADHD did not alter the conclusions in any way. Inclusion criteria for the ASD group were a formal diagnosis of ASD made by a multidisciplinary team in a standardized way according to DSM-IV-TR or DSM-5 criteria [2] and a total *T*-score above 60 on the Social Responsiveness Scale (SRS parent version [16]). Seven participants with ASD took medication to reduce symptoms related to ASD and/or ADHD (Rilatine, Concerta, Aripiprazol). The TD sample comprised healthy volunteers, matched on age, verbal and performance IQ. Parents of the TD children also completed the SRS questionnaire to exclude the presence of substantial ASD symptoms. Descriptive statistics for both groups are displayed in Table 1, showing that they did not differ for age and IQ. Evidently, both groups differed highly significantly on SRS scores.

### General procedure

The Medical Ethical Committee of the university hospital approved the study, and the participants as well as their parents provided informed consent according to the Declaration of Helsinki. All participants received a monetary reward and a small present of their choice. The experiment was embedded in a larger research project consisting of three testing sessions. Intellectual abilities were assessed in a separate session. The current frequency-tagging experiment was included in the third session.

### IQ measures

An abbreviated version of the Dutch Wechsler Intelligence Scale for Children, Third Edition (WISC-III-NL; [49, 102]) was administered. Performance IQ was estimated by the subtests Block Design and Picture Completion, verbal IQ by the subtests Vocabulary and Similarities [83].

### Stimuli

**Fig. 1:**
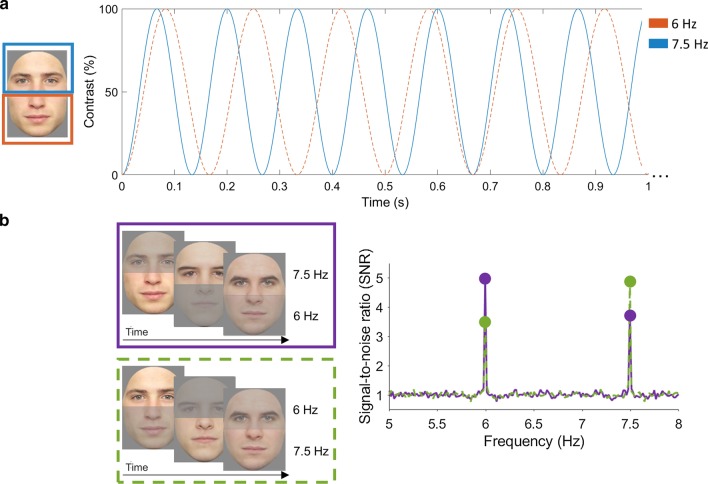
**a** Illustration of a sequence. The total experiment consisted of 4 sequences of 60 s. In each sequence, the upper and lower part of faces were presented through sinusoidal contrast-modulation (0–100%). In the illustrated example, the upper face part was presented at 7.5 Hz, while the lower face part was presented at 6 Hz. We counterbalanced frequencies (6 and 7.5 Hz). **b** Illustration of the two presented conditions with their respective SNR spectra, averaged across all participants from both groups. In the first (purple) condition, the upper face part elicits a neural response at 7.5 Hz and the lower face part at 6 Hz. In the second condition (green), the upper face part elicits a neural response at 6 Hz and the lower part elicits a neural response at 7.5 Hz. For simplicity, here, only the fundamental frequencies are shown. For neural responses at the harmonics, see Fig. 2. SNR is shown for the occipital region of interest

Twelve full-front color pictures of faces were used (stimuli from [52]). The stimuli were divided into a top and bottom face part by cutting each face horizontally in half (Fig. 1). Shown at a distance of 60 cm and at a resolution of 1920 × 1200, each face subtended a visual angle of approximately 13° in height and 6.5° in width. All faces were aligned at the nasion. A black rectangular outline surrounded the entire face image.

### Procedure

After electrode placement, participants were seated in a comfortable chair at a viewing distance of 60 cm and were instructed to maintain a constant distance. Stimuli were displayed on the screen (24-in. LED-backlit LCD monitor) through sinusoidal contrast modulation on a light grey background using Java. We used a screen with a refresh rate of 60 Hz, ensuring that the refresh rate was an integer multiple of the presentation frequencies. A sequence lasted 64 s, including 60 s of stimulation at full contrast, flanked by 2 s of fade-in and fade-out, with contrast gradually increasing and decreasing between 0 and 100%. Fade-in and fade-out were used to avoid abrupt eye movements and eye blinks due to the sudden appearance or disappearance of flickering stimuli. In total, there were four sequences; hence, the total duration of the stimulus presentation was about 4 min. In two sequences, images of female faces were shown and in the other two male faces were shown. Six different faces were presented throughout a sequence, with the identity of a face changing after a variable 8-to-12 s presentation window.

Figure 1 illustrates a stimulation sequence, consisting of the simultaneously presented top and bottom halves of a face. In each sequence, the top and bottom halves of the face stimuli flickered at different frequencies. Specifically, the two face parts were sinusoidally contrast-modulated, one at 6 Hz and the other one at 7.5 Hz. A trial started with both top and bottom face parts at zero contrast (i.e., invisible). The flicker frequencies were counterbalanced across trials and were selected so that they are close to each other and so that they could not be associated with large differences in absolute EEG response [9, 60, 73].

Participants were instructed to look freely at the images on the screen and to press a key whenever they detected brief (300 ms) changes in the color of the rectangular outline surrounding the entire face images. These color changes occurred randomly, 15 times per sequence. This task was orthogonal to the effect/manipulation of interest and ensured that participants maintained a constant level of attention throughout the entire experiment.

### EEG recording

EEG was recorded using a BioSemi Active-Two amplifier system with 64 Ag/AgCl electrodes. During recording, the system uses two additional electrodes for reference and ground (CMS, common mode sense, and DRL, driven right leg). Horizontal and vertical eye movements were recorded using four electrodes placed at the outer canthi of the eyes and above and below the right orbit. The EEG was sampled at 512 Hz.

### EEG analysis

#### Preprocessing

All EEG processing was performed using Letswave 6 (https://www.letswave.org/) and Matlab 2017 (The Mathworks). EEG data were segmented in 67-s segments (2 s before and 5 s after each sequence), bandpass-filtered (0.1–100 Hz) using a fourth-order Butterworth filter, and down-sampled to 256 Hz. Next, electrodes were visually inspected, and noisy electrodes were linearly interpolated from the 3 spatially nearest electrodes (not more than 5% of the electrodes, i.e., 3 electrodes, were interpolated). All data segments were re-referenced to a common average reference. While in frequency-tagging studies we typically apply blink correction (using ICA) for any participant blinking more than 2 standard deviations above the mean (e.g., [25, 92], [96]), in the present study we did not perform any blink correction as none of the participants blinked excessively, i.e., more than two standard deviations above the mean across all participants (0.36 times per second). Note that frequency-tagging EEG yields responses with a high SNR at specific frequency bins, while blink artefacts are broadband and thus do not generally interfere with the responses at the predefined frequency [73]. Hence, blink correction (or removal of trials with many blinks) is not systematically performed in such studies (e.g., [33, 78, 108]).

#### Frequency-domain analysis

Preprocessed segments were further cropped to contain an integer number of 1.5 Hz cycles (i.e., largest common divisor of both 6 and 7.5 Hz), beginning after fade-in and until 59.38 s (15,203 time bins). The resulting segments were averaged per condition (i.e., segments with the same combination of stimulus category and presentation rate) in the time domain to preserve the complex phase of the response and reduce EEG activity out of phase with the stimulation (i.e., noise). The averaged waveforms were transformed into the frequency domain using a fast Fourier transform (FFT), and the amplitude spectrum was computed with a high spectral resolution (0.017 Hz, 1/59.38 s), resulting in a very high signal-to-noise ratio (SNR) [60, 73].

The recorded EEG contains a signal at frequencies that are integer multiples (harmonics) of the frequencies at which images are presented (6 Hz and 7.5 Hz) (e.g., 6 Hz, 12 Hz, 18 Hz; 7.5 Hz, 15 Hz, 22.5 Hz, etc.). We used two measures to describe the response in relation to the noise level: signal-to-noise ratio (SNR) to better visualize the data (e.g., [52]) and baseline-corrected amplitudes [75] to quantify it. SNR spectra were computed for each electrode by dividing the value at each frequency bin by the average value of the 20 neighboring frequency bins (12 bins on each side, i.e., 24 bins, but excluding the 2 bins directly adjacent and the 2 bins with the most extreme values). We computed baseline-corrected amplitudes in a similar way by subtracting the average amplitude of the 20 surrounding bins. For group visualization of topographical maps (Fig. 3), we computed across-subjects averages of the baseline-corrected amplitudes for each condition and electrode separately.

Since the neural response is typically distributed over multiple harmonics, and all the harmonic frequencies represent some aspect of the periodic response, we combine the response amplitudes across all those harmonics whose response amplitude is significantly higher than the amplitude of the surrounding noise bins (as recommended in Retter and Rossion [75]; see also Rossion et al. [79]). To define the harmonics that were significantly above noise level, we computed Z-score spectra on group-level data for each stimulation frequency [25, 41, 52, 80]. We averaged the FFT amplitude spectra across electrodes in the relevant regions-of-interest (ROIs) based on topographical maps and transformed these values into Z-scores (i.e., the difference between the amplitude at each frequency bin and the mean amplitude of the corresponding 20 surrounding bins, divided by the SD of the amplitudes in these 20 surrounding bins). For 6 Hz, Z-scores were significant (i.e., *Z* > 2.32 or *p* < 0.01) until the 5th harmonic (30 Hz), and for 7.5 Hz, Z-scores until the fourth harmonic (30 Hz) were significant. We excluded the shared harmonic 30 Hz and summed the baseline-corrected amplitudes of the significant harmonics for each frequency and each condition separately. Hence, we quantified neural responses to the upper and the lower parts at 6 Hz and at 7.5 Hz by summing the baseline-subtracted responses for the significant harmonics: 6 Hz, 12 Hz, 18 and 24 Hz for the 6-Hz stimulation frequency; and 7.5 Hz, 15 Hz and 22.5 Hz for the 7.5-Hz stimulation frequency. Therefore, we obtained an index of neural saliency per stimulus type (i.e., upper vs. lower part) and per presentation rate.

Based on visual inspection of the topographical maps of both groups (Fig. 3), we identified a region of interest (ROI) in which the signal was maximal and averaged the signal at these nearby electrodes. The analysis of the response to both types of stimuli focused on a *medial occipital* ROI including Oz, Iz, O1 and O2 (Fig. 3).

#### Statistical analysis

We analyzed the baseline-corrected amplitudes in the ROI at each presentation frequency for each face part at the group level using a Bayesian general linear mixed model, relying on the R (v 4.0.2, [18]) package brms [11]. brms is a software package providing a convenient front end to STAN where Bayesian models are fitted using Hamiltonian MCMC methods [13]. In Bayesian general linear mixed models, one has to explicitly put prior distributions on the model parameters. For the parameters associated with the fixed effects, we used a normal distribution prior with mean 0 and standard deviation 1. For the standard deviation parameters associated with the random effects, we used a half-normal distribution prior with mean 0 and standard deviation 2. For the residual standard deviation, we used a half-normal prior with mean 0 and standard deviation 2. We used 4 MCMC chains each including 8000 iterations of which 4000 were considered warm-up, resulting in 16,000 posterior samples across all 4 chains. The sampling algorithm was NUTS (the No-U-Turn Sampler variant of Hamiltonian Monte Carlo). Compared to the frequentist mixed-effects models, these priors effectively act as some form of regularization ensuring parameter estimates stay within reasonable bounds. The data were analyzed with a Bayesian general linear mixed model using the neural responses (i.e., baseline-subtracted amplitudes) as dependent variables. *Face part* (upper vs. lower) as a within-subject factor and *Group* (ASD vs. TD) as a between-subject factor. We included a random intercept and a random slope for face part per participant in the model. The model we fitted can be viewed as a “full” model (including two main effects and an interaction) that (in an ANOVA framework) would be compared to “reduced” models to assess the presence/absence of main and/or interaction effects (through likelihood ratio tests or comparing AIC/BIC). As Bayesian model comparison is a complex enterprise and no simple implementation exists to assess main and interaction effects, we relied here on the parameter estimates of the full model to answer our questions of interest. That is, we summarized the posterior distributions through various pairwise comparisons comparing, for example whether proportional looking time was higher for TD vs ASD (for the main effect of group), or differed between upper and lower face parts (for the main effect of face part), etc. In this way, we assess the presence of main effects and/or an interaction effect. We report 95% posterior credible intervals on these pairwise comparisons to assess the presence or the absence of an effect.

In addition, we determined for each participant whether the response to each face part was significantly larger than the surrounding noise. The procedure was as follows (e.g., [25, 75, 96]): (1) the raw FFT amplitude spectrum was averaged across electrodes in the ROI, (2) cut into segments centered on the target frequency bin and harmonics (i.e., 6, 12, 18 and 24 Hz or 7.5, 15, 22.5 Hz), surrounded by 20 neighboring bins on each side, (3) the amplitude values across the segments of FFT spectra were summed, (4) the summed FFT spectrum was transformed into a *z*-score using the 20 surrounding bins (see above). Responses of a given participant were considered significant if the z-score at the target frequency bin exceeded 1.64 (i.e., *p* < 0.05 one-tailed: signal > noise).

### Eye-tracking recording

Eye-tracking data were collected using a Tobii X3-120 screen-based remote eye tracker and Tobii Pro software (Tobii Pro). The sampling rate was 120 Hz. Binocular gaze precision at ideal conditions is estimated at 0.24° of visual angle and binocular gaze accuracy at 0.4°. However, for many experiments these ideal conditions are not met [59]. With a remote eye tracker, participants are free to move their head within the “headbox” allowing eye tracking [59]. Due to this freedom of movement, as well as the use of pediatric and/or patient populations, the precision and accuracy of the actual data may differ from those marketed by the manufacturers. In the standard calibration procedure of the Tobii X3-120, participants have to follow a red dot moving across the screen, yielding a merely qualitative index of calibration quality based on visual inspection. In order to obtain a subject-specific *quantitative* measure of eye-tracking data quality, we implemented an additional calibration validation paradigm, preceding the data registration. In this additional calibration procedure, participants had to fixate on the center of nine consecutive fixation crosses appearing on different locations on the screen. Calculation of the angle between the vectors to the displayed fixation cross versus the actual gaze point yields a quantitative index of error angle (mean and variance) and resulting accuracy. These values were used in the analysis to attribute gaze points more accurately to particular areas of interest (AOIs). For two participants (one from the ASD group and one from the TD group), eye-tracking data were not recorded due to technical failure.

### Eye-tracking analysis

In short, the eye-tracking analysis involved the assignment of fixations to predefined areas of interest (AOIs), calculation of the proportional looking times for each of the AOIs, and modeling of the temporal face exploration dynamics along these AOIs.

#### Fixation filter, definition of areas of interest (AOI), gaze attribution

Eye-tracking data were analyzed by means of a series of custom-built MATLAB (Matlab 2019b, The Mathworks) scripts (see https://osf.io/mv45x/). We used the I2MC algorithm (identification by 2-means clustering [37]), to filter the raw eye-tracking data (i.e., deleting random noise, interpolating missing data, identifying fixations). In the current study, the three AOIs (eyes, mouth, and nose) were defined using the limited-radius Voronoi tessellation (LRVT), as it has been shown to be the most noise-robust and objective method for defining AOIs for facial stimuli [36]. In addition, we defined the area “outside AOI” to comprise all the fixation points that were not assigned to either of the AOIs.

Fixations are attributed to the AOIs by means of a probability weighting, taking into account the subject-specific data quality as obtained via the additional calibration validation procedure. For every gaze point, a proportional score between zero and one is attributed to every AOI (i.e., “eyes,” “mouth,” “nose” and “outside face”), in such a way that the cumulative sum of these scores equals one. The size of this score indicates the probability that the corresponding AOI effectively contains the recorded gaze point, taking into account the subject-specific data quality. Assignment of proportional scores depends on a two-dimensional bell curve around the gaze point with a standard deviation equal to the root mean square (RMS) registered during calibration validation. Hence, the calibration validation determines the probability weighting of the AOIs: better data quality results in more concentrated sample points around the gaze point, and poorer data quality results in more dispersed sample points. Since the algorithm takes every gaze point into account, as well as the data quality, it proves to be a reliable method. We used the same method in [98]. For each AOI, the relative duration of all fixation points was averaged over the four trials.

#### Statistical analysis

We analyzed the eye-tracking data using a Bayesian general linear mixed model, relying on the R package brms [11]. Similarly to the statistical analysis of the EEG data, we used a normal distribution prior with mean 0 and standard deviation 1 for the parameters associated with the fixed effects. For the standard deviation parameters associated with the random effects, we used a half-normal distribution prior with mean 0 and standard deviation 2. For the residual standard deviation, we used a half-normal prior with mean 0 and standard deviation 2. We used 4 MCMC chains each including 8000 iterations of which 4000 were considered warm-up, resulting in 16,000 posterior samples across all 4 chains. The sampling algorithm was NUTS (the No-U-Turn Sampler variant of Hamiltonian Monte Carlo). The proportional looking time was examined with a Bayesian general linear mixed model using *area of interest* (*eyes, mouth, nose*) as a within-subject factor, and *group* (ASD vs. TD) as a between-subject factor. We included a random intercept and a random slope for face part per participant in the model. Further, the analysis approach was identical to the one applied for the EEG data. We relied on the parameter estimates of the full model to answer our questions of interest. We summarized the posterior distributions through various pairwise comparisons comparing, for example, whether proportional looking time was higher for TD vs ASD (for the main effect of group), or differed between eyes, mouth and nose (for the main effect of area of interest), etc. In this way, we assess the presence of main effects and/or an interaction effect. We report 95% posterior credible intervals on these pairwise comparisons to assess the presence or the absence of an effect.

#### Correlations between EEG, eye tracking and Social Responsiveness Scales

To investigate the relationship between the frequency-tagging EEG, the eye-tracking responses and the Social Responsiveness Scales, we calculated Spearman’s rank correlation coefficients (Corrplot package in R [103]. *P* values were FDR-corrected for multiple comparisons.

#### Face exploration dynamics

We modeled the temporal dynamics of the gazing behavior using Observable Markov Models (OMMs), using a custom python implementation. The OMMs consist of a transition matrix T, where element T_ij_ represents the probability of finding a fixation point in AOI j at time *t* + 1, given that the fixation point at *t* falls inside AOI i. The Markov property assumes this transition probability to be independent of the previous states. To estimate the elements of the transition matrix T, we counted all transitions from AOI i to AOI j for each participant. In a preprocessing step, we interpolated the data using a second-order spline fit. For each participant, we constructed a single transition matrix.

We retained the fixation data falling within the four AOIs (left eye, right eye, nose, mouth) and removed fixations outside of these AOIs by constructing a limited radius Voronoi tessellation (with a maximum radius equivalent to 100 pixels, see Additional file 1: Figure S1). Additionally, to remove the data of participants gazing at the edge of the image or outside the image, we limited the dataset to those fixation data falling inside the face image, by applying a margin of 50 pixels from the mean point of the AOI (see vertical cutoff in Additional file 1: Figure S1).

To fully characterize the face exploration dynamics, in addition to the OMM transition probabilities, we computed the mean fixation duration, the mean saccadic amplitude and the mean intraparticipant dispersion [20]. The mean fixation duration and mean saccadic amplitude are obtained by averaging the fixation durations and saccadic amplitudes, respectively, that are given in the output of Tobii Pro, for the data falling inside the four AOIs. The intraparticipant dispersion is defined as the mean Euclidean distance between all data within a trial.

The statistical significance of the difference in dynamic scanning patterns between the ASD and TD group is computed using a MANOVA test on the OMM transition matrix elements and the three additional face exploration features. We remove the last column of the transition matrix, since these elements induce multicollinearity due to the normalization condition (each row sums to 1). Hence, we constructed a property vector for each participant containing the 12 entries of the transition matrix, the mean fixation duration, the mean saccadic amplitude, and the mean intraparticipant dispersion. Accordingly, the resulting vectors each contained 15 features characterizing the dynamic gazing behavior of a participant. Next, all 15 entries were group-independently (across participants) standardized to a distribution with mean 0 and standard deviation of 1, to remove any dependency on the chosen scales. As a result, the actual property vectors contained the z-scores of the respective features. Finally, to account for the strong correlation between these 12 features, we carried out a group-independent principal component analysis (PCA), with an 0.98 explained variance ratio cutoff. The latter corresponds to keeping 13 principal components, using the default values of the scikit-learn package (v 0.22.1, [65]), resulting in the retention of 13 orthogonal principal components. Based on the local outlier factor method, two participants (from the ASD group) were detected as outliers and were consequently removed from the MANOVA group comparison analysis.

For the individual-level classification analysis, we opted for a linear discriminant analysis (LDA), applied to the 15 data features. We chose LDA since the model is simple and gives a robust estimate even without tuning any hyperparameters.

## Results

### No group difference in orthogonal task performance

We did not observe group differences on the behavioral color change detection task, suggesting a similar level of attention throughout the experiments. This result is in line with previous frequency-tagging studies comparing school-aged boys with ASD and typically developing boys [93, 96, 98]. Both groups showed accuracies between 95 (SD = 0.6%) and 97.7% (SD = 0.1%) with mean response times between 0.49 (SD = 0.008) and 0.46 (SD = 0.002) seconds, for ASD and TD, respectively. Statistical analyses (two-sided *t*-tests) showed no significant differences between the ASD group and the TD group (accuracy: *t*(38) = − 1.25, *p* = 0.11; response times *t*(38) = 1.32, *p* = 0.097).

### No reduced saliency for eyes or increased saliency for mouth in neural responses in ASD

**Fig. 2:**
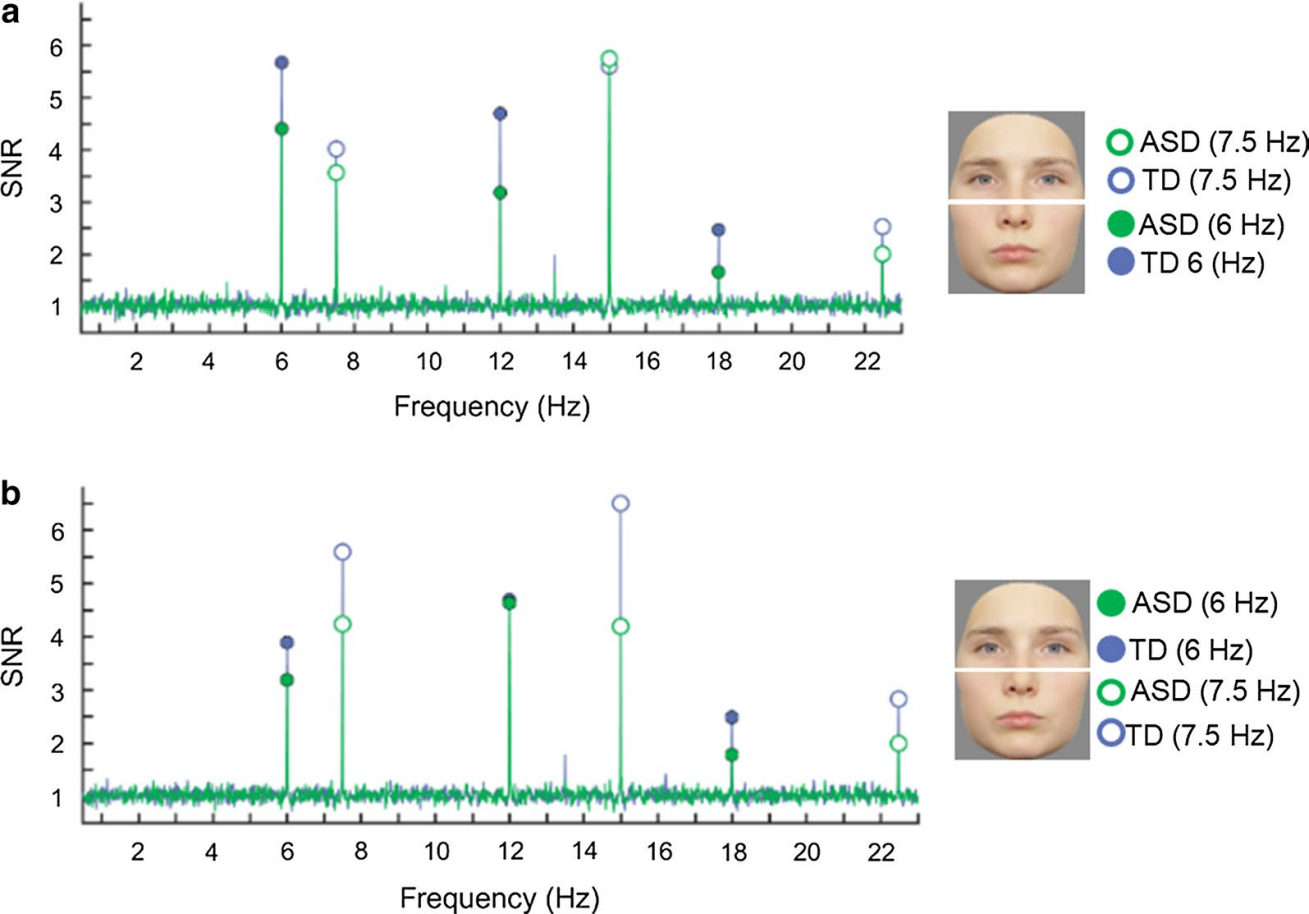
Signal-to-noise ratio (SNR) EEG spectra of the different conditions, averaged over the electrodes of the occipital region of interest (O1, O2, Iz, Oz). In **a**, the lower face part was presented at 6 Hz (filled circles) and the upper part at 7.5 Hz (open circles), whereas in **b** the lower face part was presented at 7.5 Hz (open circles) and the upper part at 6 Hz (filled circles). The frequencies were counterbalanced. Clear SNR peaks can be observed at the frequencies of interest (and harmonics)

**Fig. 3:**
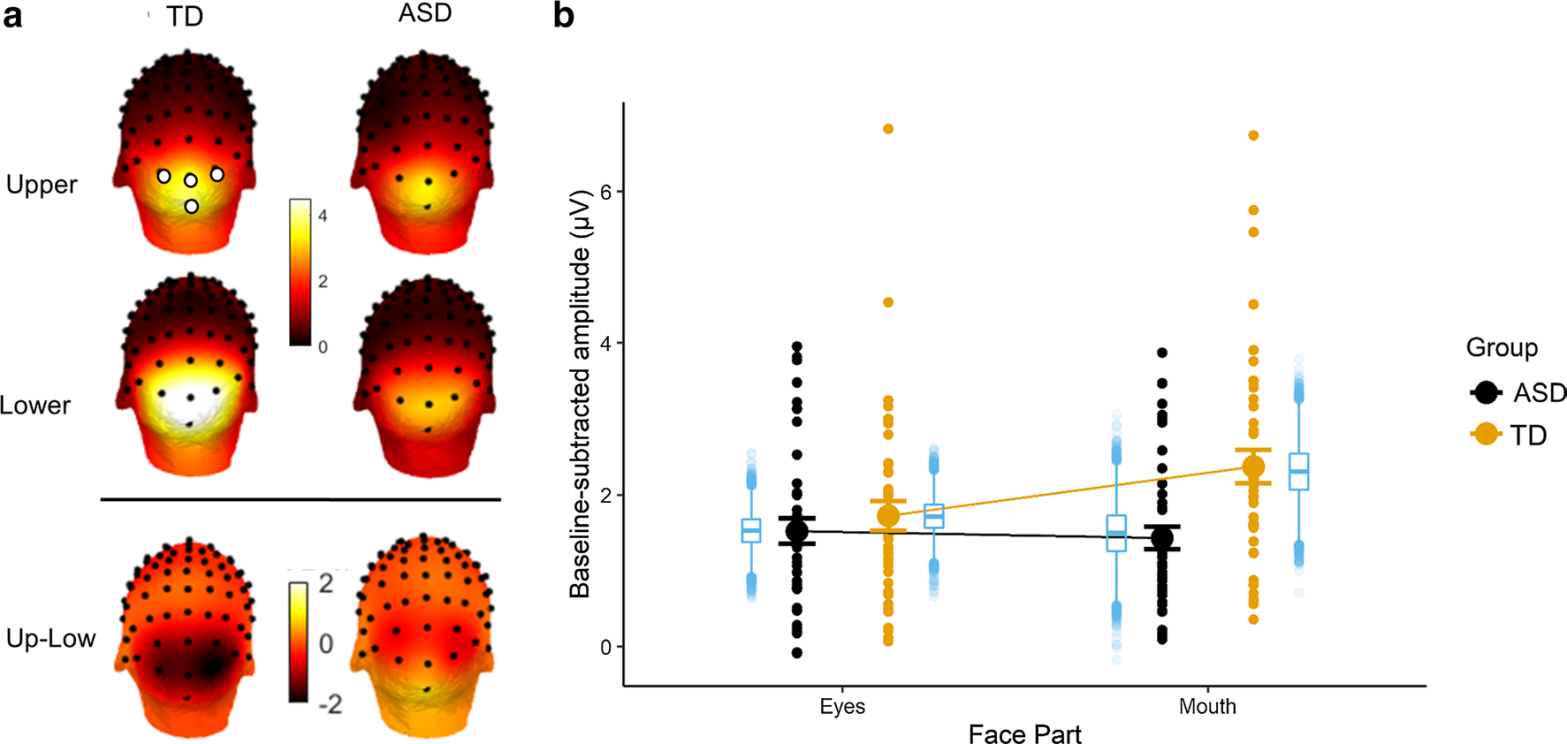
**a** Scalp distribution of the EEG signal during frequency-tagging (baseline subtracted amplitudes in µV). Frequency-tagged neural responses to the periodically presented face parts are shown for each group, as well as the differential response for “upper” minus “lower” face part. The analysis of the response to both types of stimuli focused on an occipital region of interest (O1, O2, Iz, Oz), indicated with the open circles in the upper left scalp topography. **b** Averaged baseline-subtracted amplitudes for each stimulus condition (upper face part versus lower face part) for each group. The mean, SEM and the individual subject data are displayed in black and orange for the ASD and TD group, respectively. In blue, boxplots depict means and interquantile ranges of the posterior estimates of the Bayesian model

**Fig. 4:**
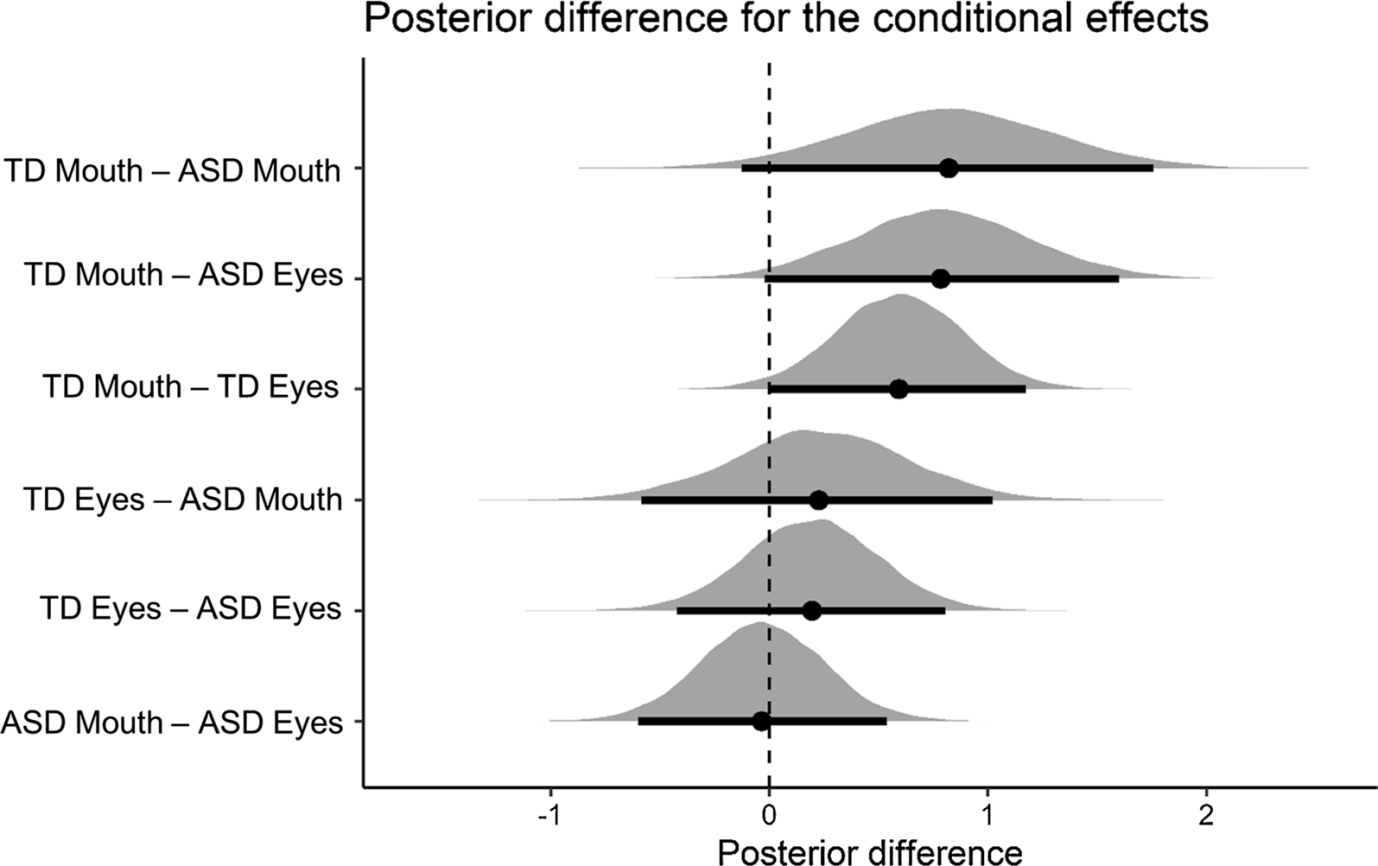
Visualizations of the posterior distributions of the effects (pairwise comparisons) of the neural EEG responses. Black dots show the posterior mean of the conditional effect; horizontal bars and lines denote the 95% posterior credible intervals. On the *Y*-axis, effects are sorted from the largest to the smallest posterior difference. An effect is considered significant if 0 does not lie within the 95% credible interval of the posterior difference. For example, in the first line, TD mouth—ASD mouth presents the posterior distribution of the difference between the neural response of the TD group and the ASD group to the mouth. For this condition effect, the 95% credible interval contains 0. Therefore, we are not able to reject the null hypothesis that there is no difference between the conditions

We observed frequency-tagged EEG responses for the two face halves in the medial occipital region of interest (ROI) (see Fig. 2 for SNR, Fig. 3 for scalp distributions and averaged response amplitudes and Fig. 4 for posterior differences of the effects). Analyses at the individual level indicated that despite the short recording time, all participants showed significant responses to the upper and the lower face halves.

At the group level, statistical analyses showed no significant main effect of *Group.* Averaged across both face parts, participants in the TD group had slightly larger EEG responses than participants in the ASD group (TD: 2.01 µV, ASD: 1.46 µV; 95% CI of the difference = [− 1.33; 0.17]). No significant main effect of *Face Part* was observed (upper part: 1.59 µV, lower part: 1.88 µV; 95% CI of the difference = [− 0.72; 0.15]).

Considering the interaction effects between *Group* and *Face part*, there was no significant difference in the amplitude of the neural responses for the upper part in the TD group (1.68 µV) as compared to the ASD group (1.50 µV) (95% CI of the difference = [− 0.85; 0.45]), nor for the lower part (TD = 2.34 µV, ASD = 1.42 µV; 95% CI of the difference = [− 2; 0.081]).

Breaking up the interaction in the other way (i.e., differences between *Face Parts* within *Group*), participants from the ASD group did not have significantly larger neural responses to any of the face parts (upper part = 1.50 µV, lower part = 1.42 µV, 95% CI of the difference = [− 0.52; 0.72]). In the TD group, the neural response to the lower part (2.34 µV) is significantly larger than the response to the upper part (1.68 µV) (95% CI of the difference = [0.03; 1.28]).

Finally, we computed the pairwise differences between each pairwise *interaction effect* (Is the difference between face parts in the TD group stronger than in the ASD group?). The differences between *face parts* did not differ between groups (95% CI of the difference = [− 0.135; 1.64]), indicating that there is no evidence that the patterns of neural responses are different between groups.

### Both groups look more to the eyes than the mouth and proportional looking times to these parts do not differ between groups

Analysis of the eye tracking data quality reveals slight differences between groups. The average error angle was significantly higher in the ASD group (0.56° ± 0.06°) than in the TD group (0.40° ± 0.046°) (*t*(39) = − 2.14, *p* = 0.038). The root mean square of this angle, however, did not differ between groups (ASD: 0.68 ± 0. 19, TD: 0.64 ± 0.63; *t*(39) = − 0.24, *p* = 0.41).

**Fig. 5:**
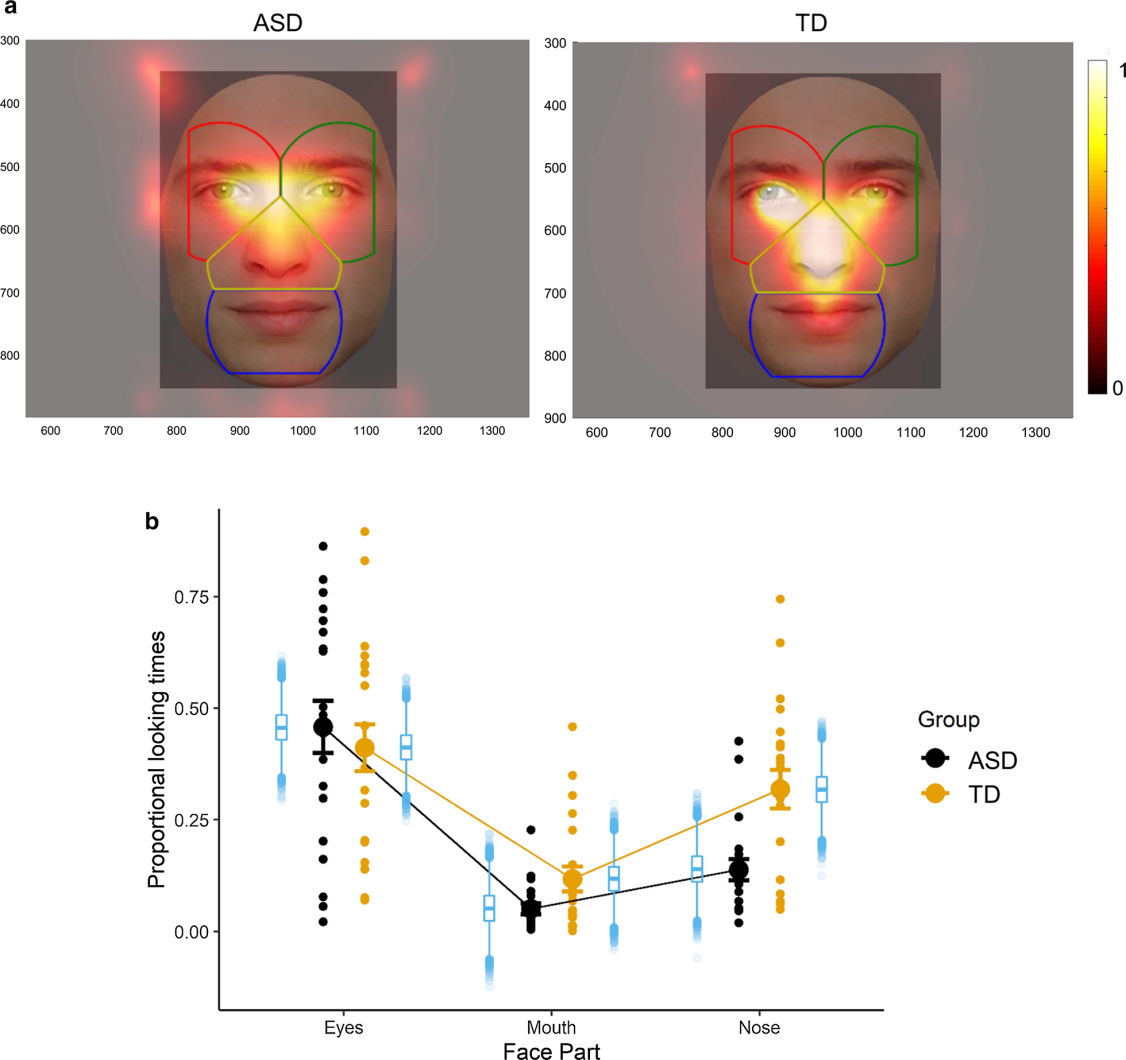
**a** Visualization of the Voronoi AOI tessellation on an example face stimulus. Heatmaps of looking time averaged over all participants of the ASD group (left) and the TD group (right). The heatmap depicts the looking time at a certain pixel relative to the maximal looking time. **b** The mean, SEM and the individual subject data of the proportional looking times to the facial features in each group are displayed in black and orange for the ASD and TD group, respectively. In blue, boxplots depict means and interquantile ranges of the posterior estimates of the Bayesian model

Both participant groups looked relatively often outside the face AOIs (Fig. 5 and Additional file 1: Figure S1). This proportional looking time outside the face was different between groups: TD: 16%, SEM = 0.036; ASD: 35%, SEM = 0.063, *t*(39) = 2.81, *p* = 0.008.

**Fig. 6:**
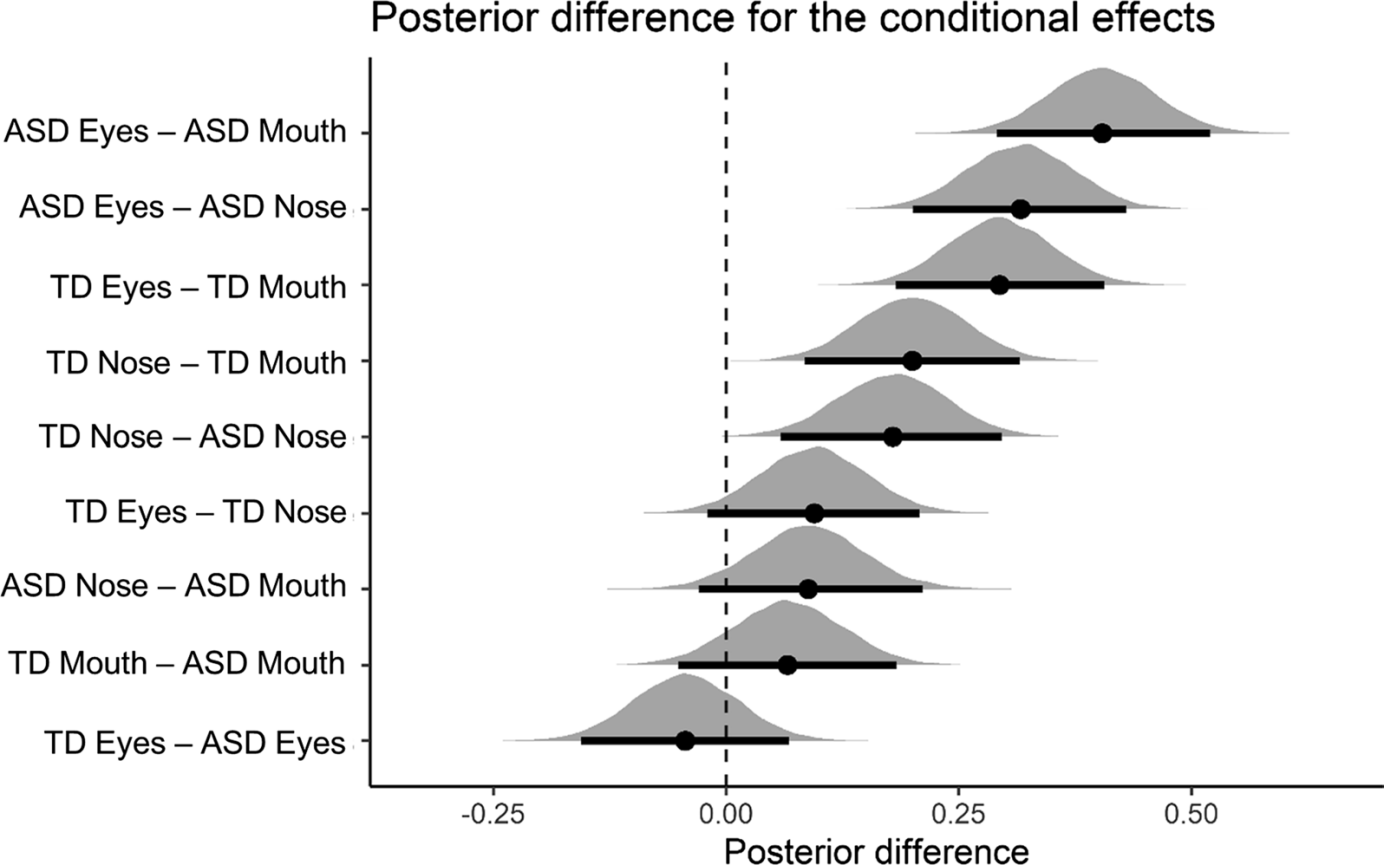
Visualizations of the posterior effects of the eye-tracking data. Black dots show the posterior mean of the conditional effect; horizontal bars and lines denote the 95% posterior credible intervals. An effect is considered significant if 0 does not lie within the 95% credible interval of the posterior difference. The interpretation of the figure is similar to Fig. 4

Results of the Bayesian mixed model analysis are presented in Figs. 5 and 6. Regarding the gaze points falling in the face AOIs, the Bayesian analysis revealed that there was no main effect of group. Averaged across all face parts, participants in the TD group had slightly higher proportional looking times than participants in the ASD group (TD: 28.3%, ASD: 21.7%; 95% CI of the difference = [− 13.4%; 0.001%]). A main effect of area of interest was observed. That is, across groups, the eyes were looked at more (43.4%) than the mouth (8.5%) (95% CI of the difference = [26.9%; 43%]). The eyes were also looked at more than the nose (23.1%; 95% CI of the difference = [12.4%; 28.7%]). Third, the nose was looked at more than the mouth (95% CI of the difference = [6.3%; 22.6%]).

Considering the interaction effects between group and face part, there was no difference in the amount of time spent looking at the eyes in the TD group (41.2%) as compared to the ASD group (45.8%) (95% CI of the difference = [− 6.5%; 15.8%]), nor for looking at the mouth (TD: 11.8%; ASD: 5.1%; 95% CI of the difference = [− 18.1%; 4.7%]). There was a significant group difference in the amount of time spent looking at the nose (TD: 31.8%; ASD: 13.8%; 95% CI of the difference = [6.6%; 29.8%]).

Breaking up the interaction in the other way (i.e., differences between face parts within group), participants from the ASD group looked significantly more to the eyes (45.8%) than to the nose (13.9%) (95% CI of the difference = [20.5%, 43.4%]). This effect was not significant in the TD group (eyes: 41.1%, nose: 31.8%, 95% CI of the difference = [− 1.9%; 20.6%]). Participants in the TD group looked more to the nose (31.8%) than to the mouth (11.8%) (95% CI of the difference = [8.6%; 31.3%]). This effect was not significant in the ASD group (nose: 13.9%, mouth: 5.1%; 95% CI of the difference = [− 20.1%; 3.2%]). Participants in the ASD group looked more to the eyes than to the mouth (95% CI of the difference = [29.1%; 51.9%]). The same pattern held for participants in the TD group (95% CI of the difference = [18.4%; 40.6%]).

Finally, as the difference between significant and nonsignificant is not necessarily significant, we computed the pairwise differences between each pairwise interaction effect (e.g., is the group difference for the eyes stronger than for the mouth?). The group differences in looking time did not differ between eyes and mouth (95% CI of the difference = [− 4.7%; 27.3%]) nor between mouth and nose (CI = [− 5%; 27.9%]), but the time spent looking at the eyes versus the nose was different between the groups (CI = [6.7%; 38.5%]).

### Face exploration dynamics differ between groups: more exploration in the ASD group

**Fig. 7:**
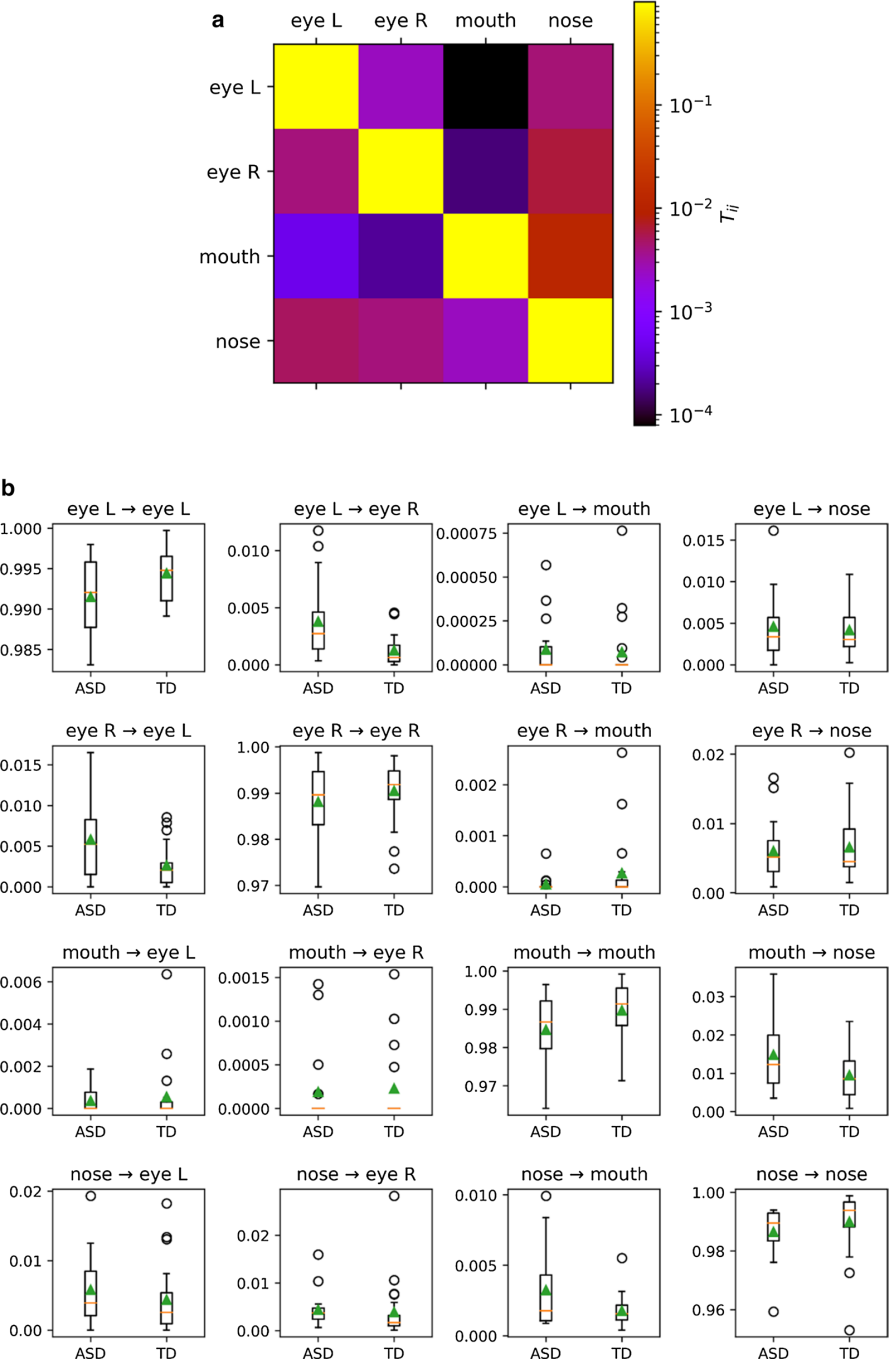
**a** Average transition matrix for all participants. The color scale is logarithmic for clarity. The values in the diagonal elements are high (almost approaching 1), indicating that there is a high chance of maintaining gaze in the same AOI**.** **b** Boxplots of the transition probabilities between the different AOIs for each group. The boxes extend from the lower to the upper quartile values. The median of each group is represented by the orange lines. Note the differences in *Y*-axis scaling

The average transition matrix (over all participants) of the OMM models is shown in Fig. 7a. This matrix indicates that in most of the subsequent time steps, the fixation point remains within the same AOI. This can be observed in the probabilities of the elements along the diagonal, which are close to 1. As can be expected, the transition from eyes to mouth and vice versa is mainly reached by first passing through the nose AOI, which is reflected in T (eyes → mouth) close to 0. Furthermore, we observe that it is more common to transition from the nose to the eyes than from the nose to the mouth AOI. This is in agreement with the observation from Fig. 5 that the proportional looking time to the mouth AOI is generally low.

**Fig. 8:**
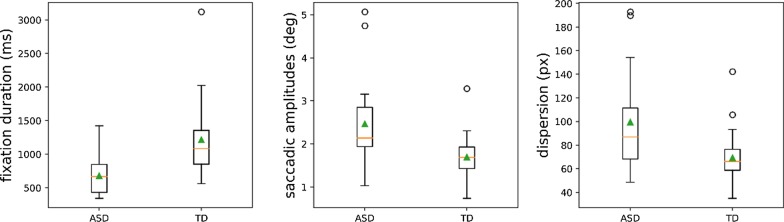
Boxplots of fixation duration, saccadic amplitudes, and the intra-participant dispersion per group (across all AOIs)

In Fig. 7b, each of these transition probabilities is depicted for the TD and the ASD group separately. Here, the most important group difference is situated along the diagonal, where we observe that the probability to maintain a gaze inside the same AOI in subsequent time steps is higher in the TD group than in the ASD group. Conversely, from the off-diagonal elements, one can observe that the probability to transition between different AOIs is higher in the ASD group as compared to the TD group. Similar effects are found in Fig. 8, showing that fixation duration is larger in the TD group, whereas saccadic amplitude and intra-participant dispersion are higher in the ASD group.

More specifically, we observe in the upper left block diagonal of Fig. 7b (containing the four T(eye → eye) elements) that in the TD group the probability to maintain a gaze inside the same AOI in subsequent time steps is higher than for the ASD group. In the ASD group, the probability to transition between the eyes in subsequent time steps is higher compared to the TD group. Next, we consider the third row, containing the conditional probabilities to transition from the mouth to the four AOIs. Again, we observe that the gaze of the TD group has a higher probability of staying within the mouth AOI, whereas in the ASD group chances are higher to transition to another AOI. The last row contains the conditional probability to transition from the nose to the four AOIs and shows that again the TD group keeps their gaze fixed, whereas the ASD group tends to transition to other AOIs.

In summary, these results suggest that the ASD group displays a more exploratory dynamic gaze behavior compared to the TD group. To statistically test whether the dynamic gazing behavior is significantly different between the two groups, we carried out a MANOVA. First, to handle the strong correlations in the data (of the 15 features: 12 Markov transition elements, and 3 additional measures), a PCA reduction is applied. The results of this MANOVA confirm that there is a significant difference in face exploration scan paths between the ASD and the TD group (*F*(12,28) = 3.1, *p* = 0.022).

To determine the individual separability based on the OMM approach (with the three additional measures), we constructed a classification model using linear discriminant analysis (LDA) on the z-scores of the 15 original features (not the PCA components). We find a classification accuracy of 72% based on a leave-one-out analysis (with trivial accuracy 53.8%). This implies that there is 72% chance to correctly predict group membership based on the face exploration dynamics. To determine the statistical significance of this result, we ran the leave-one-out classification test 10,000 times through a permutation test. Hereby, in each permutation run, we randomly permuted the labels and performed the leave-one-out validation. Each single classification includes standardizing the data features (by determining the mean and standard deviation on the training set) and fitting the LDA model on the training set. We obtained *p* = 0.006, implying that the chance to obtain at least 72% percent correct classification with random labels is 0.6%.

### EEG and eye tracking are correlated

In general, over the full trial length, the proportional looking times at the eyes and the amplitude of the frequency-tagged EEG response for the upper face half were significantly correlated in both groups (TD: *ρ* = 0.51, *p* = 0.01; ASD: *ρ* = 0.61, *p* = 0.01) and indicates that individuals who look more to the eyes also show increased neural responses for the face half with the eyes. Likewise, proportional looking times at the mouth and the EEG response to the lower half were significantly correlated in the TD group (*ρ* = 0.58, *p* = 0.01). However, in the ASD group, this association did not reach significance (*ρ* = 0.2, *p* = 0.71). This implies that the neural responses for the mouth region are only marginally driven by the time spent looking at the mouth region in the ASD group. In the ASD group, looking more outside the face was significantly associated with looking less to the eyes (*ρ* = − 0.79, *p* < 0.001) and the nose (*ρ* = − 0.69, *p* = 0.02), as well as lower EEG responses to the eyes (*ρ* = − 0.72, *p* < 0.001) and the mouth (*ρ* = − 0.56, *p* = 0.01). Looking at the nose and EEG, responses for the mouth were significantly correlated in the ASD group (*ρ* = 0.66, *p* = 0.01). Note that the nose AOI is situated at the interface of the upper and lower flickering face halves. These correlations were not significant in the TD group. In the TD group, looking more to the nose was significantly correlated with lower EEG responses to the upper half (*ρ* = − 0.52, *p* = 0.02).

#### Correlations between task measures and Social Responsiveness scale

In the TD group, but not the ASD group, we found a significant correlation between looking outside the facial AOIs and a higher total SRS score (*ρ* = 0.68, *p* < 0.001). In none of the groups, we observed significant correlations between the Social Responsiveness Scale, and proportional looking times for the eyes or the mouth, or neural responses to the upper vs lower part of the face (all *p* > 0.05).

## Discussion

### No evidence for group differences in proportional looking times and neural responses to eye vs. mouth region

Often, people with ASD do not make the type of eye contact others generally expect. These gaze abnormalities have typically been studied with eye tracking, allowing the measurement of overt visual attention via fixation recordings. The present study was motivated by the fact that eye tracking precludes measuring the processing of stimuli outside the focus of overt visual attention. Thus, in addition to measuring overt processes with eye tracking, we used frequency-tagging EEG to study covert face processing in TD and ASD boys. Importantly, this frequency-tagging approach allows monitoring the intensity of neural responses to multiple stimuli, also those that are outside the overt attentional locus. Changes in the EEG amplitude of the markers represent dynamic neural changes related to the processing of the driving stimulus. More specifically, we presented images of the upper and the lower parts of the face at 6 Hz and 7.5 Hz or vice versa to quantify the neural saliency of processing the eye region versus the mouth region of a face.

Overall, classical eye-tracking measures (proportional looking times) and neural responses showed no support for the “excess mouth/diminished eye gaze” hypothesis in ASD. In particular, pertaining to the looking behavior, both groups looked longer to the eyes than to the mouth, and we did not observe group differences in fixation duration to these features. Remarkably, pertaining to the neural data, the EEG pattern was largely unexpected. Firstly, because in the TD participants, the lower face part with the mouth evoked significantly larger neural responses than the upper part with the eyes, even though individuals looked more to the eye region than the mouth region. Secondly, because differences in neural saliency between the upper part with the eyes and the lower part with the mouth did not emerge for the ASD group, they were present for the TD group. Finally, pertaining to the association between looking behavior and neural responses, we observed that individual differences in looking times to the upper face half were significantly correlated with individual differences in frequency-tagged EEG response to the same face halve in both groups. Likewise, increased looking time to the mouth region was associated with larger EEG response to the lower face half, but this association did only reach significance in the TD group.

In spite of the popularity of the “excess mouth/diminished eye gaze” hypothesis in ASD, we did not find any empirical evidence for it. This lack of empirical evidence is in line with several individual studies and meta-analyses on preferential looking in ASD (e.g., [27, 30, 31, 50, 64]), which all found little support for this appealing hypothesis. As mentioned in the introduction, across these studies, looking behavior and inconsistent findings have been related to several factors, such as the degree of symptom severity, the use of small and heterogeneous participant samples, differences in outcome measures, differences in stimulus and task characteristics, etc. Likewise, our eye-tracking findings may be determined by several factors inherent to the experiment or the participant group. First, our experiment involved the presentation of neutral faces without external features such as hair and ears. While this type of stimuli ascertains control over low-level stimulus properties, they are not highly ecologically valid. It is possible that the use of other, more realistic face stimuli (e.g., embedded within a naturalistic scene) or expressive faces would have yielded different results. Second, it is known from the earliest eye-tracking studies that task demands and context influence gaze behavior [106]. Hence, by using active face processing tasks or by using stimuli displaying social interactions that necessitate particular gaze behavior, we might have observed different results. Third, the use of a well-selected, well-matched and homogeneous participant sample in terms of age, gender, IQ and diagnostic status ensured that any observed differences between groups, if found, would be related to ASD symptomatology and not to other factors. On the other hand, however, one may question whether the current findings may generalize to other samples or whether other samples may have revealed group differences in face processing. Nevertheless, in spite of all these potentially shaping factors, the absence of evidence for the “excess mouth/diminished eye gaze” hypothesis in ASD in our study adds to a growing list of studies, suggesting that this is not a valid hypothesis. In this regard, it is important to note that multiple recent reviews concluded that the empirical evidence for the “excess mouth/diminished eye gaze” hypothesis in ASD is very limited, even while using more realistic and dynamic scenes across multiple participant samples (ranging from infants to adults and from low-functioning to high-functioning individuals) (e.g., [27, 30, 31, 50, 64]). Thus, even though abnormalities in the social use of eye contact constitute one of the key clinical symptoms of ASD (DSM-5; [2]), this should not necessarily imply that it applies to all individuals with the diagnosis. Moreover, it is only an assumption driven by an initial pioneering study [48] that abnormal use of eye contact in daily life would manifest in laboratory situations as decreased looking to the eyes and increased looking to the mouth. In this regard, the recent movement of using real-life models in research might shed light on this issue (e.g., [12, 71]). For example, it has been shown that in neurotypicals, direct eye contact with a real-life model increased skin conductance (a proxy for stress arousal) more strongly as compared to direct gaze from a video-recorded model [71].

The reason why eye contact may be atypical in (some) individuals with ASD is currently unknown. It is probable that the experience of eye contact varies across the autism population. ASD reflects a heterogeneous spectrum, so some individuals with ASD may be indifferent to eye contact, whereas others may experience it as unpleasant or intrusive. Many autism therapies encourage children and adults to make eye contact. To design the most optimal intervention approach, it is important to understand whether clinicians are teaching people with ASD to pay attention to something that does not interest them or that makes them feel uncomfortable. While we could not fully answer this question, our study with the combined eye tracking and EEG approach suggests that looking to the eyes is not particularly uncomfortable or atypical for the ASD group. In the first place, because participants with ASD spend a similar amount of time looking to the eye region. Secondly, because here, we demonstrate that individuals with ASD show a very similar neural EEG response to this specific eye region. If looking at the eyes would have been irrelevant or highly uncomfortable for individuals with ASD, a respective lower or higher EEG response would have been expected, even when the proportional looking time is identical. One could argue that the identical neural response toward eye gaze in the ASD group may result from combining a subgroup of indifferent low responders with a subgroup of oversensitive high responders, but this hypothetical pattern is not supported by inspection of the group variances nor the individual subject data. Accordingly, based on the current EEG data obtained with frequency-tagged neutral faces, we have to conclude that direct gaze is not experienced any differently in boys with ASD. This finding aligns with a study investigating the modulation of the autonomic nervous system (ANS) response based on direct gaze, showing similar modulation of pupillometry responses in ASD as in controls ([62], but see [45]). Evidently, as is the case in most face processing research, the current study merely involved the processing of fairly artificial non-interactive still face stimuli, which does not preclude that more robust group differences in the (neural) processing of eye contact may still be observed in real-life dyadic interactions.

### Larger neural responses for lower vs. upper face halves in TD participants

Despite looking more to the eye region than to the mouth region, TD participants showed larger neural responses for the lower halve of the face displaying the mouth region. Several factors can account for this observation. First, the total surface of the face that is frequency-tagged is slightly larger for the bottom face half than the top half (about 3.5%). Second, there may be more physical similarity between the different top halves than the bottom face halves appearing during a stimulation sequence. Third, there may be an advantage in processing shapes in the lower versus upper visual field [84]. Finally, TD participants looked a large amount of time at the nose region, which can explain this pattern. Across all participants, looking at the nose is related to larger neural responses to the mouth (note that the correlation does not reach significance in the TD group, presumably due to the few data points). Moreover, the TD controls look lower in the nose AOI, closer to the mouth (Fig. 5). Together, these results indicate that while individual differences in the amplitude of the neural responses are partially determined by the fixations, frequency-tagged neural responses additionally measure covert attention. This is also illustrated in a study by de Vries and Baldauf [24]: While fixation remained constant (on the nasion), top-down attention to either the top or the bottom half of faces increased the frequency-tagged MEG response to that respective part. Moreover, by combining these responses with fMRI, they found that frequency-tagged neural responses to the mouth were mostly measured over the occipital face area (OFA), whereas responses to the eyes were measured in the OFA and the superior temporal sulcus (STS). In the current study, responses to both face halves were maximal over occipital areas, possibly suggesting that the STS activity to the eyes was not captured by the scalp electrodes, leading to lower responses to the upper face half with the eyes.

### Atypical eye contact in ASD is reflected by more exploratory face scanning style

By probing the individual temporal patterns in the eye-tracking data, we observed significant group differences in face exploration dynamics that did predict individual group membership with an accuracy of 72%, *p* = 0.06. The individual face exploration dynamics involved fixation transition matrices obtained from observable Markov models (OMM), the mean fixation duration, saccadic amplitude and dispersion. Specifically, we observed that the ASD group used a more exploratory and less stable face scanning strategy as compared to the TD group. These results contradict previous studies finding less exploration and more visual persistence in ASD [32, 76, 91]. However, in these other studies, gaze exploration was investigated in dynamic scenes, in face-to-face interactions or in pictures of everyday scenes. In our study, in contrast, static and isolated faces were presented without external features and without particular task demands. These characteristics may have potentially limited explorative behavior in general, as can also be observed in Fig. 7, in which the values along the diagonal are large. In line with our finding of more gaze exploration in the ASD group, other studies also found reduced fixation durations in school-aged children with ASD and 6–9-month-old infants at high risk for ASD as compared to TD controls [43, 46, 57, 101]. While we observed significant group differences in face exploration, the accuracy of assigning group membership is only moderate. Especially when base rate occurrence of ASD is taken into account [1 in 59 children according to the most recent estimates of the Center for Disease Control (CDC; [4]; but see [29]), there is not much evidence that one would be able to provide a powerful screening instrument based on these features. Still, these results underline the value of taking the temporal dynamics of eye-tracking data into account. For example, the fact that participants from the ASD group shift their gaze more frequently as compared to TD controls might suggest atypical eye contact, even when total looking time at the eyes is the same. Since this is the first study using OMMs to probe facial dynamics in ASD, further studies should assess the generalizability of this result to other stimuli and participants.

The observation that participants in the ASD group switch more frequently between different AOIs might relate to the more feature-based face perception strategy often reported in ASD, in contrast to the configural or holistic face processing strategy employed by neurotypical individuals (e.g., [8]). Participants in the TD group look mostly at the eyes and nose, and they generally keep fixating the same AOI. This point between the eyes and the nose is related to the ‘optimal/preferred fixation point’ for a range of face perception tasks [67] and has also been shown to be the preferred first fixation point in free social interactions [69]. Participants from the ASD group generally look a bit higher around the eyes, and they tend to switch more often between the facial features, possibly reflecting a less configural and more feature-based perceptual style. A more feature-based and less holistic face perception strategy was also observed in a previous EEG study with this same sample of participants [96]. There we found a strong face inversion effect for implicit facial identity discrimination in the TD group, while this inversion effect was absent in the ASD group, pointing toward a less configural and more feature-based face perceptual style.

### Overt vs. covert attention to faces

Participants from the ASD group also look more outside the face. This finding is in line with the literature, for instance with other findings [5, 50, 61, 98]. This might reflect the fact that not particularly the eyes, but the whole face in general is perceived as less attractive or even threatening and therefore avoided. Although the neural responses are not in line with this interpretation (no group difference in neural responses), it might be that participants from the ASD group slightly look next to the face, at the edges. (This can also be seen in Fig. 5.) So overtly they might not look at the faces, but covertly they still seem to process the faces to the same extent. In this study, a rectangle surrounded the face images and participants were instructed to press a button when the color of the rectangle changed. Although behavioral results were not different between groups, participants of the ASD group might have been slightly more focused on this orthogonal task.

### Limitations

This study initiates new questions that will require further research. In particular, whether these results are generalizable to a broader range of different participants should be investigated. Here, we only included a relatively small, selective subsample of boys with ASD showing average to above-average intelligence and no language deficits. Due to the small sample size, particularly the discriminative analyses should be regarded as exploratory. With small sample sizes, discriminative analyses are prone to overfitting if one does not pay sufficient attention to the complexity of the model. Therefore, models that do not require extensive (hyper-) parameter fine-tuning should be preferred, and permutation tests should be implemented to assess the statistical significance of the result. Although permutation testing ensured statistical control, future work should validate the analyses in independent samples. Although the homogeneity of this sample allowed controlling for confounding factors such as age, IQ and gender, future studies may benefit from the inclusion of females with ASD and more severely affected individuals, including individuals with low IQ. The implicit and fast nature of the experiment makes it extremely applicable to different participant samples that are otherwise not easy to test.

## Conclusions

Despite finding differential proportional looking times toward the eyes or the mouth of a face and in the saliency of neural processing of the two regions of a face as determined by frequency-tagging EEG, the present study did not report group differences between boys with and without ASD. This suggests that the eye region is not experienced as less relevant (which would be indexed by lower saliency) or more threatening (which would be indexed by increased saliency) in ASD. Together with the intact eye-tracking data, these findings refute the excess mouth/diminished eye-processing hypothesis in ASD. Modeling of the individual temporal dynamics of the eye gaze data demonstrates that boys with ASD apply a more explorative face exploration strategy, possibly reminiscent of the more feature-based and less configural perceptual style, which has often been suggested in ASD.

## Supplementary information

**Additional file 1**. Figure S1. Fixation data of all participants. As an illustration, using color, we differentiate between the four AOI regions and data falling outside the AOIs (purple). Fixations out of these AOIs were removed by constructing a limited radius Voronoi partitioning (with a maximum radius of 100 pixels). Additionally, to remove the effects of participants gazing at the edge of the image or outside the image, we limited the dataset to those data falling inside the face image, where a margin of 50 pixels is taken into account.

## Data Availability

No part of the study procedures or analysis plans was preregistered in an institutional registry prior to the research being conducted. The conditions of our ethics approval do not permit public archiving of individual anonymized raw data. Readers seeking access to the data should contact the lead author Sofie Vettori and/or the senior author Bart Boets at the Center for Developmental Psychiatry, Department of Neurosciences, University of Leuven. Access will be granted to named individuals in accordance with ethical procedures governing the reuse of sensitive data. Specifically, requestors must complete a formal data sharing agreement to obtain the data. Stimuli and analysis scripts can be found in https://osf.io/mv45x/.

## Abbreviations

ASD: Autism spectrum disorder
AOI: Area of interest (eye tracking)
EEG: Electroencephalography
FFT: Fast Fourier transform
FPVS: Fast periodic visual stimulation
OFA: Occipital face area
ROI: Region of interest (EEG)
STS: Superior temporal sulcus
TD: Typically developing

## References

[1] Adrian ED, Matthews BHC (1934). The interpretation of potential waves in the cortex. J Physiol.

[2] American Psychiatric Association (2013). Diagnostic and statistical manual of mental disorders (DSM-5).

[3] Andersen SK, Fuchs S, Müller MM (2011). Effects of feature-selective and spatial attention at different stages of visual processing. J Cogn Neurosci.

[4] Baio J, Wiggins L, Christensen DL, Maenner MJ, Daniels J, Warren Z (2018). Prevalence of autism spectrum disorder among children aged 8 years—autism and developmental disabilities monitoring network, 11 sites, United States, 2014. MMWR Surveill Summ..

[5] Bal E, Harden E, Lamb D, Van Hecke AV, Denver JW, Porges SW (2010). Emotion recognition in children with autism spectrum disorders: relations to eye gaze and autonomic state. J Autism Dev Disord.

[6] Barton JJS, Radcliffe N, Cherkasova MV, Edelman J, Intriligator JM (2006). Information processing during face recognition: the effects of familiarity, inversion, and morphing on scanning fixations. Perception.

[7] Blais C, Jack RE, Scheepers C, Fiset D, Caldara R (2008). Culture shapes how we look at faces. PLoS ONE.

[8] Behrmann M, Thomas C, Humphreys K (2006). Seeing it differently: visual processing in autism. Trends Cogn Sci.

[9] Boremanse A, Norcia AM, Rossion B (2014). Dissociation of part-based and integrated neural responses to faces by means of electroencephalographic frequency tagging. Eur J Neurosci.

[10] Buchan JN, Paré M, Munhall KG (2007). Spatial statistics of gaze fixations during dynamic face processing. Soc Neurosci.

[11] Bürkner PC (2017). brms: An R package for Bayesian multilevel models using Stan. J Stat Softw.

[12] Cañigueral R, Ward JA, Hamilton AFDC (2020). Effects of being watched on eye gaze and facial displays of typical and autistic individuals during conversation. Autism.

[13] Carpenter B, Gelman A, Hoffman MD, Lee D, Goodrich B, Betancourt M, Brubaker M, Guo J, Li P, Riddell A. Stan: A probabilistic programming language. J Stat Softw. 2017;76(1). 10.18637/jss.v076.i01 PMC9788645 36568334

[14] Chawarska K, Shic F (2009). Looking but not seeing: atypical visual scanning and recognition of faces in 2 and 4-year-old children with autism spectrum disorder. J Autism Dev Disord.

[15] Chevallier C, Kohls G, Troiani V, Brodkin ES, Schultz RT (2012). The social motivation theory of autism. Trends Cogn Sci.

[16] Constantino JN, Gruber CP (2012). Social responsiveness scale (SRS).

[17] Corden B, Chilvers R, Skuse D (2008). Avoidance of emotionally arousing stimuli predicts social-perceptual impairment in Asperger’s syndrome. Neuropsychologia.

[18] Core Team, R (2020). R: a language and environment for statistical computing.

[19] Coutrot A, Binetti N, Harrison C, Mareschal I, Johnston A (2016). Face exploration dynamics differentiate men and women. J Vis.

[20] Coutrot A, Hsiao JH, Chan AB (2018). Scanpath modeling and classification with hidden Markov models. Behav Res Methods.

[21] Critchley HD, Daly EM, Bullmore ET, Williams SC, Van Amelsvoort T, Robertson DM, Rowe A, Phillips M, McAlonan G, Howlin P, Murphy DG (2000). The functional neuroanatomy of social behaviour: changes in cerebral blood flow when people with autistic disorder process facial expressions. Brain.

[22] Dalton KM, Nacewicz BM, Johnstone T, Schaefer HS, Gernsbacher MA, Goldsmith HH, Alexander AL, Davidson RJ (2005). Gaze fixation and the neural circuitry of face processing in autism. Nat Neurosci.

[23] Dapretto M, Davies MS, Pfeifer JH, Scott AA, Sigman M, Bookheimer SY, Iacoboni M (2006). Understanding emotions in others: mirror neuron dysfunction in children with autism spectrum disorders. Nat Neurosci.

[24] de Vries E, Baldauf D (2019). Attentional weighting in the face processing network: a magnetic response image-guided magnetoencephalography study using multiple cyclic entrainments. J Cogn Neurosci.

[25] Dzhelyova M, Jacques C, Rossion B (2017). At a single glance: fast periodic visual stimulation uncovers the spatio-temporal dynamics of brief facial expression changes in the human brain. Cereb Cortex.

[26] Falck-Ytter T, Fernell E, Gillberg C, von Hofsten C (2010). Face scanning distinguishes social from communication impairments in autism. Dev Sci.

[27] Falck-Ytter T, von Hofsten C (2011). How special is social looking in ASD: a review. Prog Brain Res.

[28] Fletcher-Watson S, Leekam SR, Findlay JM, Stanton EC (2008). Brief report: young adults with autism spectrum disorder show normal attention to eye-gaze information-evidence from a new change blindness paradigm. J Autism Dev Disord.

[29] Fombonne E (2018). The rising prevalence of autism. J Child Psychol Psychiatr..

[30] Frazier TW, Strauss M, Klingemier EW, Zetzer EE, Hardan AY, Eng C, Youngstrom EA (2017). A meta-analysis of gaze differences to social and nonsocial information between individuals with and without autism. J Am Acad Child Adolesc Psychiatry.

[31] Guillon Q, Hadjikhani N, Baduel S, Rogé B (2014). Visual social attention in autism spectrum disorder: insights from eye tracking studies. Neurosci Biobehav Rev.

[32] Heaton TJ, Freeth M (2016). Reduced visual exploration when viewing photographic scenes in individuals with autism spectrum disorder. J Abnorm Psychol.

[33] Hemptinne C, Liu-Shuang J, Yuksel D, Rossion B (2018). Rapid Objective assessment of contrast sensitivity and visual acuity with sweep visual evoked potentials and an extended electrode array. Invest Ophthalmol Vis Sci.

[34] Henderson JM, Williams CC, Falk RJ (2005). Eye movements are functional during face learning. Mem Cogn.

[35] Hessels RS (2020). How does gaze to faces support face-to-face interaction? A review and perspective. Psychon Bull Rev.

[36] Hessels RS, Kemner C, van den Boomen C, Hooge ITC (2016). The area-of-interest problem in eyetracking research: a noise-robust solution for face and sparse stimuli. Behav Res Methods.

[37] Hessels RS, Niehorster DC, Kemner C, Hooge ITC (2017). Noise-robust fixation detection in eye movement data: identification by two-means clustering (I2MC). Behav Res Methods.

[38] Hillyard SA, Anllo-Vento L (1998). Event-related brain potentials in the study of visual selective attention. Proc Natl Acad Sci.

[39] Hopfinger JB, Luck SJ, Hillyard SA (2004). Selective attention: electrophysiological and neuromagnetic studies. Cogn Neurosci.

[40] Hsiao JH, Cottrell G (2008). Two fixations suffice in face recognition. Psychol Sci.

[41] Jacques C, Retter TL, Rossion B (2016). A single glance at natural face images generate larger and qualitatively different category-selective spatio-temporal signatures than other ecologically-relevant categories in the human brain. NeuroImage.

[42] Jones W, Carr K, Klin A (2008). Absence of preferential looking to the eyes of approaching adults predicts level of social disability in 2-year-old toddlers with autism spectrum disorder. Arch Gen Psychiatry.

[43] Joseph RM, Keehn B, Connolly C, Wolfe JM, Horowitz TS (2009). Why is visual search superior in autism spectrum disorder?. Dev Sci..

[44] Joseph RM, Tanaka J (2003). Holistic and part-based face recognition in children with autism. J Child Psychol Psychiatry.

[45] Kaartinen M, Puura K, Mäkelä T, Rannisto M, Lemponen R, Helminen M, Salmelin R, Himanen S-L, Hietanen JK (2012). Autonomic arousal to direct gaze correlates with social impairments among children with ASD. J Autism Dev Disord..

[46] Kemner C, Verbaten MN, Cuperus JM, Camfferman G, Van Engeland H (1998). Abnormal saccadic eye movements in autistic children. J Autism Dev Disord..

[47] Kliemann D, Dziobek I, Hatri A, Steimke R, Heekeren HR (2010). Atypical reflexive gaze patterns on emotional faces in autism spectrum disorders. J Neurosci.

[48] Klin A, Jones W, Schultz R, Volkmar F, Cohen D (2002). Visual fixation patterns during viewing of naturalistic social situations as predictors of social competence in individuals with autism. Arch Gen Psychiatry.

[49] Kort W, Schittekatte M, Dekker PH, Verhaeghe P, Compaan EL, Bosmans M, Vermeir G, Derde Editie N (2005). WISC-III NL wechsler intelligence scale for children. Handleiding en Verantwoording.

[50] Kwon M-K, Moore A, Barnes CC, Cha D, Pierce K (2019). Typical levels of eye-region fixation in toddlers with autism spectrum disorder across multiple contexts. J Am Acad Child Adolesc Psychiatry.

[51] Lewkowicz DJ, Hansen-Tift AM (2012). Infants deploy selective attention to the mouth of a talking face when learning speech. Proc Natl Acad Sci USA.

[52] Liu-Shuang J, Norcia AM, Rossion B (2014). An objective index of individual face discrimination in the right occipito-temporal cortex by means of fast periodic oddball stimulation. Neuropsychologia.

[53] Mehoudar E, Arizpe J, Baker CI, Yovel G (2014). Faces in the eye of the beholder: unique and stable eye scanning patterns of individual observers. J Vis.

[54] Morgan ST, Hansen JC, Hillyard SA (1996). Selective attention to stimulus location modulates the steady-state visual evoked potential. Proc Natl Acad Sci USA.

[55] Moriuchi JM, Klin A, Jones W (2017). Mechanisms of diminished attention to eyes in autism. Am J Psychiatry.

[56] Müller MM, Andersen S, Trujillo NJ, Valdés-Sosa P, Malinowski P, Hillyard SA (2006). Feature-selective attention enhances color signals in early visual areas of the human brain. Proc Natl Acad Sci USA.

[57] Nackaerts E, Wagemans J, Helsen W, Swinnen SP, Wenderoth N, Alaerts K (2012). Recognizing biological motion and emotions from point-light displays in autism spectrum disorders. PloS One..

[58] Nakano T, Tanaka K, Endo Y, Yamane Y, Yamamoto T, Nakano Y, Ohta H, Kato N, Kitazawa S (2010). Atypical gaze patterns in children and adults with autism spectrum disorders dissociated from developmental changes in gaze behaviour. Proc Biol Sci.

[59] Niehorster DC, Cornelissen THW, Holmqvist K, Hooge ITC, Hessels RS (2018). What to expect from your remote eye-tracker when participants are unrestrained. Behav Res Methods.

[60] Norcia AM, Appelbaum LG, Ales JM, Cottereau BR, Rossion B (2015). The steady-state visual evoked potential in vision research: a review. J Vis.

[61] Nuske HJ, Vivanti G, Hudry K, Dissanayake C (2014). Pupillometry reveals reduced unconscious emotional reactivity in autism. Biol Psychol.

[62] Nuske HJ, Vivanti G, Dissanayake C (2015). No evidence of emotional dysregulation or aversion to mutual gaze in preschoolers with autism spectrum disorder: an eye-tracking pupillometry study. J Autism Dev Disord..

[63] Ogai M, Matsumoto H, Suzuki K, Ozawa F, Fukuda R, Uchiyama I, Suckling J, Isoda H, Mori N, Takei N (2003). FMRI study of recognition of facial expressions in high-functioning autistic patients. NeuroReport.

[64] Papagiannopoulou EA, Chitty KM, Hermens DF, Hickie IB, Lagopoulos J (2014). A systematic review and meta-analysis of eye-tracking studies in children with autism spectrum disorders. Soc Neurosci.

[65] Pedregosa F, Varoquaux G, Gramfort A, Michel V, Thirion B, Grisel O, Blondel M, Prettenhofer P, Weiss R, Dubourg V, Vanderplas J, Passos A, Cournapeau D, Brucher M, Perrot M, Duchesnay É (2011). Scikit-learn: machine learning in Python. J Mach Learn Res.

[66] Pelphrey KA, Sasson NJ, Reznick JS, Paul G, Goldman BD, Piven J (2002). Visual scanning of faces in autism. J Autism Dev Disord.

[67] Peterson MF, Eckstein MP (2012). Looking just below the eyes is optimal across face recognition tasks. Proc Natl Acad Sci USA.

[68] Peterson MF, Eckstein MP (2013). Individual differences in eye movements during face identification reflect observer-specific optimal points of fixation. Psychol Sci.

[69] Peterson MF, Lin J, Zaun I, Kanwisher N (2016). Individual differences in face-looking behavior generalize from the lab to the world. J Vis.

[70] Pierce K, Müller RA, Ambrose J, Allen G, Courchesne E (2001). Face processing occurs outside the fusiform “face area” in autism: evidence from functional MRI. Brain.

[71] Prinsen J, Alaerts K (2019). Eye contact enhances interpersonal motor resonance: comparing video stimuli to a live two-person action context. Soc Cogn Affect Neurosci.

[72] Regan D (1966). Some characteristics of average steady-state and transient responses evoked by modulated light. Electroencephalogr Clin Neurophysiol.

[73] Regan D (1989). Human brain electrophysiology: evoked potentials and evoked magnetic fields in science and medicine.

[74] Regan D, Heron JR (1969). Clinical investigation of lesions of the visual pathway: a new objective technique. Journal of neurology, neurosurgery, and psychiatry.

[75] Retter TL, Rossion B (2016). Uncovering the neural magnitude and spatio-temporal dynamics of natural image categorization in a fast visual stream. Neuropsychologia.

[76] Rigby SN, Stoesz BM, Jakobson LS (2016). Gaze patterns during scene processing in typical adults and adults with autism spectrum disorders. Res Autism Spectr Disord..

[77] Rossion B (2014). Understanding face perception by means of prosopagnosia and neuroimaging. Front Biosci (Elite Ed).

[78] Rossion B, Boremanse A (2011). Robust sensitivity to facial identity in the right human occipito-temporal cortex as revealed by steady-state visual-evoked potentials. J Vis.

[79] Rossion B, Retter TL, Liu-Shuang J. Understanding human individuation of unfamiliar faces with oddball fast periodic visual stimulation and electroencephalography. Eur J Neurosci. 2020. 10.1111/ejn.14865 32542962

[80] Rossion B, Torfs K, Jacques C, Liu-Shuang J (2015). Fast periodic presentation of natural images reveals a robust face-selective electrophysiological response in the human brain. J Vis.

[81] Rutherford MD, Clements KA, Sekuler AB (2007). Differences in discrimination of eye and mouth displacement in autism spectrum disorders. Vis Res.

[82] Rutherford MD, Towns AM (2008). Scan path differences and similarities during emotion perception in those with and without autism spectrum disorders. J Autism Dev Disord.

[83] Sattler JM (2001). Assessment of children: cognitive applications.

[84] Schmidtmann G, Logan AJ, Kennedy GJ, Gordon GE, Loffler G (2015). Distinct lower visual field preference for object shape. J Vis..

[85] Speer LL, Cook AE, McMahon WM, Clark E (2007). Face processing in children with autism: effects of stimulus contents and type. Autism.

[86] Spezio ML, Adolphs R, Hurley RSE, Piven J (2007). Abnormal use of facial information in high-functioning autism. J Autism Dev Disord.

[87] Stacchi L, Liu-Shuang J, Ramon M, Caldara R (2019). Reliability of individual differences in neural face identity discrimination. NeuroImage.

[88] Tanaka JW, Sung A (2016). The “eye avoidance” hypothesis of autism face processing. J Autism Dev Disord.

[89] Thigpen NN, Bradley MM, Keil A (2018). Assessing the relationship between pupil diameter and visuocortical activity. J Vis.

[90] Toffanin P, de Jong R, Johnson A, Martens S (2009). Using frequency tagging to quantify attentional deployment in a visual divided attention task. Int J Psychophysiol.

[91] Vabalas A, Freeth M (2016). Brief report: patterns of eye movements in face to face conversation are associated with autistic traits: evidence from a student sample. J Autism Dev Disord.

[92] Van der Donck S, Dzhelyova M, Vettori S, Mahdi SS, Claes P, Steyaert J, Boets B (2020). Rapid neural categorization of angry and fearful faces is specifically impaired in boys with autism spectrum disorder. J Child Psychol Psychiatry.

[93] Van der Donck S, Dzhelyova M, Vettori S, Thielen H, Steyaert J, Rossion B, Boets B (2019). Fast periodic visual stimulation eeg reveals reduced neural sensitivity to fearful faces in children with autism. J Autism Dev Disord.

[94] Van Der Geest JN, Kemner C, Verbaten MN, Engeland HV (2002). Gaze behavior of children with pervasive developmental disorder toward human faces: a fixation time study. J Child Psychol Psychiatry.

[95] Vatikiotis-Bateson E, Eigsti IM, Yano S, Munhall KG (1998). Eye movement of perceivers during audiovisual speech perception. Percept Psychophys.

[96] Vettori S, Dzhelyova M, Van der Donck S, Jacques C, Steyaert J, Rossion B, Boets B (2019). Reduced neural sensitivity to rapid individual face discrimination in autism spectrum disorder. NeuroImage Clin.

[97] Vettori S, Dzhelyova M, Van der Donck S, Jacques C, Steyaert J, Rossion B, Boets B (2020). Frequency-tagging EEG of superimposed social and non-social visual streams reveals reduced saliency of social information in boys with autism. Front Psychiatry.

[98] Vettori S, Dzhelyova M, Van der Donck S, Jacques C, Van Wesemael T, Steyaert J, Rossion B, Boets B (2020). Combined frequency-tagging EEG and eye tracking reveal reduced social bias in boys with autism spectrum disorder. Cortex.

[99] Vialatte F-B, Maurice M, Dauwels J, Cichocki A (2010). Steady-state visually evoked potentials: focus on essential paradigms and future perspectives. Prog Neurobiol.

[100] Walker-Smith GJ, Gale AG, Findlay JM (1977). Eye movement strategies involved in face perception. Perception.

[101] Wass SV, Jones EJ, Gliga T, Smith TJ, Charman T, Johnson MH (2015). Shorter spontaneous fixation durations in infants with later emerging autism. Sci Rep..

[102] Wechsler D (1991). The Wechsler intelligence scale for children.

[103] Wei T, Simko V. R package “corrplot”: visualization of a correlation matrix; 2017. https://github.com/taiyun/corrplot.

[104] Wolf JM, Tanaka JW, Klaiman C, Cockburn J, Herlihy L, Brown C, South M, McPartland J, Kaiser MD, Phillips R, Schultz RT (2008). Specific impairment of face processing abilities in children with autism spectrum disorder using the Let’s Face It! Skills Battery. Autism Res.

[105] de Xivry J-JO, Ramon M, Lefèvre P, Rossion B (2008). Reduced fixation on the upper area of personally familiar faces following acquired prosopagnosia. J Neuropsychol.

[106] Yarbus AL. Eye movements during perception of complex objects. In: Eye movements and vision. Boston, MA: Springer; 1967. pp. 171–211.

[107] Yarbus A (1976). Eye movements and vision.

[108] Zimmermann FGS, Yan X, Rossion B (2019). An objective, sensitive and ecologically valid neural measure of rapid human individual face recognition. R Soc Open Sci.

