# Supplementary material for: Combined frequency-tagging EEG and eye-tracking measures provide no support for the “excess mouth/diminished eye attention” hypothesis in autism
Source: Mol Autism. 2020 Nov 23;11:94. doi: 10.1186/s13229-020-00396-5 (PMC7686749; doi:10.1186/s13229-020-00396-5)
Supplement: Supplementary file 1 — Additional file 1. Figure S1. Fixation data of all participants. As an illustration, using color, we differentiate between the four AOI regions and data falling outside the AOIs (purple). Fixations out of these AOIs were removed by constructing a limited radius Voronoi partitioning (with a maximum radius of 100 pixels). Additionally, to remove the effects of participants gazing at the edge of the image or outside the image, we limited the dataset to those data falling inside the face image, where a margin of 50 pixels is taken into account. [file 13229_2020_396_MOESM1_ESM.docx]

**Supplemental information**


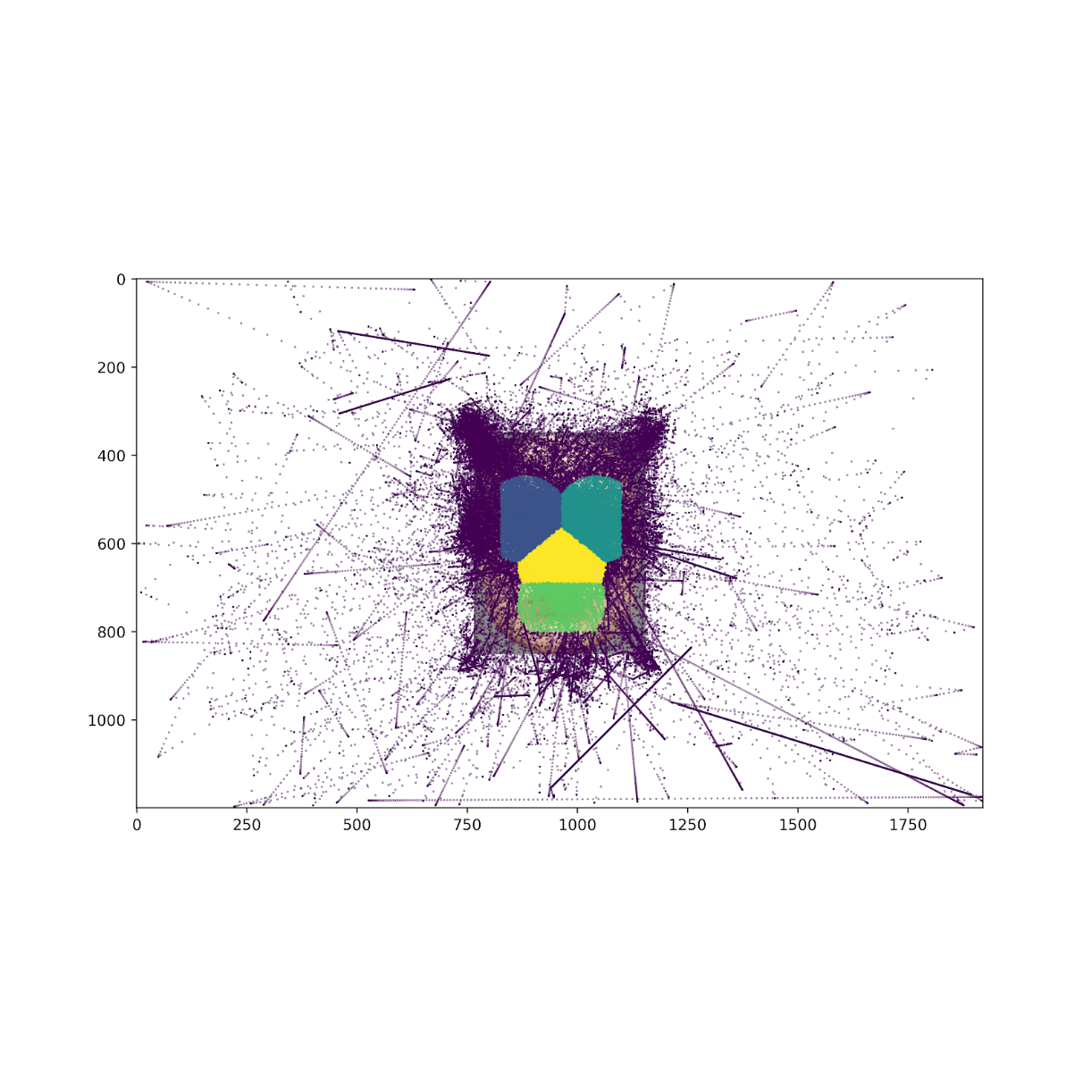


***Figure S1. Fixation data of all participants****. As an illustration, using color, we differentiate between the four AOI regions and data falling outside the AOIs (purple). Fixations out of these AOIs were removed by constructing a limited radius Voronoi partitioning (with a maximum radius of 100 pixels). Additionally, to remove the effects of participants gazing at the edge of the image or outside the image, we limited the dataset to those data falling falling inside the face image, where a margin of 50 pixels is taken into account*
